# Comparative study of decolorization processes for dyed cotton waste: enabling circularity through white feedstock

**DOI:** 10.1039/d6ra05247a

**Published:** 2026-10-21

**Authors:** Elsa Vuorenmaa, Md. Reazuddin Repon, Tonmoy Saha, Shubhajit Dutta, Inge Schlapp-Hackl, Tapani Vuorinen, Ali R. Tehrani-Bagha

**Affiliations:** a Department of Bioproducts and Biosystems, School of Chemical Engineering, Aalto University Vuorimiehentie 1 02150 Espoo Finland ali.tehrani@aalto.fi

## Abstract

The growing volume of textile waste requires effective decolorization strategies to enable recycling routes. This study compares two-bath and one-bath alkaline-reductive decolorization processes for mixed multi-colored reactive-dyed post-consumer cotton waste using sodium carbonate (Na_2_CO_3_) and sodium dithionite (Na_2_S_2_O_4_). The decolorizing agents (Na_2_CO_3_ and Na_2_S_2_O_4_) were selected based on the safe and sustainable by design (SSbD) framework. Process conditions were optimized based on colorimetric measurements, and the properties of the treated fabrics were evaluated using tensile strength retention, weight loss, intrinsic viscosity, and degree of polymerization, X-ray diffraction, scanning electron microscopy, thermogravimetric analysis, and CHNS elemental analysis. The two-bath decolorization processes achieved upto 99% decolorization and and one-bath achieved ≥97% decolorization and lightness values above 80, while maintaining high tensile strength retention and preserving the cellulose crystalline structure. Elemental analysis confirmed efficient removal of sulfur-containing dyes, and thermogravimetric analysis showed that the thermal degradation behavior of decolorized cotton closely approached that of undyed cotton. Compared with the two-bath route, the integrated one-bath process delivered higher lightness, more uniform decolorization, reduced energy and water consumption, and consequently lower wastewater treatment demand. These results demonstrate that the continuous one-bath alkaline-reductive process can provide recyclable cellulosic white feedstock from reactive-dyed cotton waste with improved process efficiency and lower environmental impact, supporting scalable textile-to-textile circularity.

## Abstract 2

Na_2_CO_3_/Na_2_S_2_O_4_ decolorization converts mixed reactive-dyed cotton waste into white feedstock for recycling. The one-bath route gives >97% color stripping, >88% strength retention and preserved crystallinity, with less water and energy.

## Graphical abstract

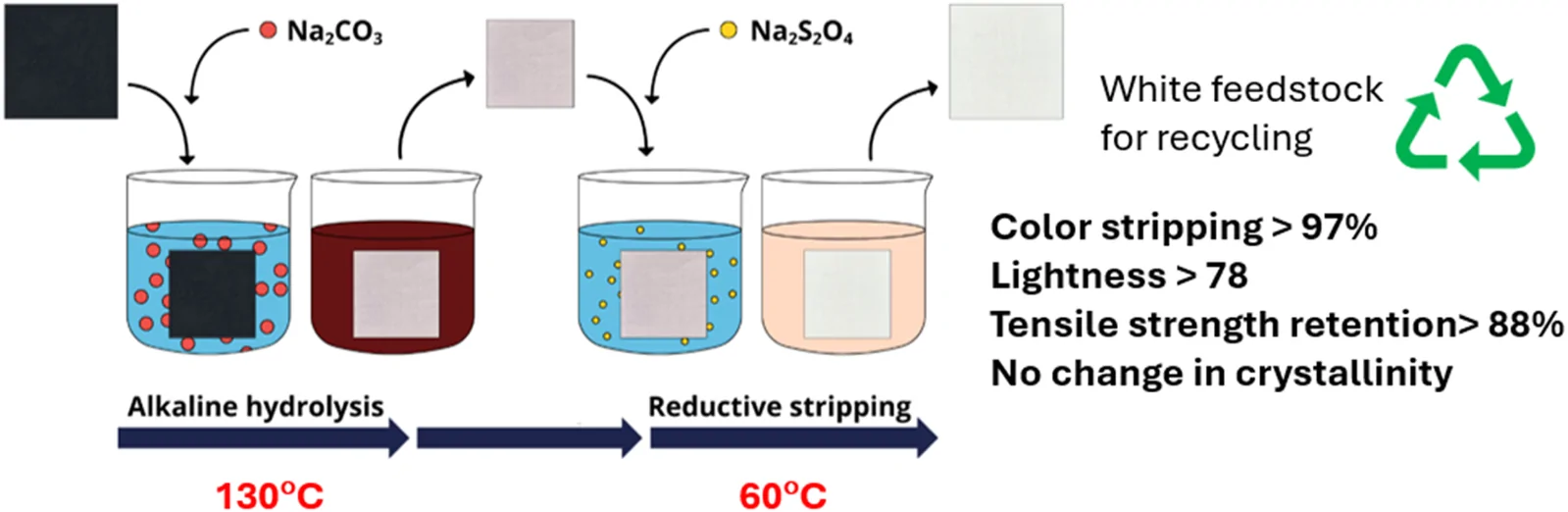

## 1 Introduction

Global textile waste is currently estimated to be approximately 120 million tons per year and projected to exceed 150 million tons by 2030. About 80% is landfilled or burned, 12% is reused, and <1% is recycled into new textiles.^1^ One of the main barriers to textile-to-textile recycling of post-consumer textiles is the presence of dyes, which cannot be reprocessed without prior decolorization.^2–4^ Cotton fiber, which is predominantly used in textile applications, is mostly dyed with reactive dyes. In particular, the removal of reactive dyes is challenging because they form covalent bonds with cellulose and their persistence can complicate the subsequent fiber-to-fiber recycling of dyed cotton waste.^5–7^

High-whiteness cotton feedstock supports the production of high-quality textiles through both mechanical and chemical recycling. Residual dyes reduce product quality, compatibility with virgin fibers, re-dyeability, and interfere with the dissolution and regeneration processes, thereby restricting textile circularity.^8,9^ Various chemical approaches have been explored to remove reactive dyes from cotton waste, including oxidative, reductive, and combined redox systems. Oxidative methods, such as H_2_O_2_ or Fenton reactions, can achieve high decolorization but often cause cellulose degradation and strength reduction.^5,10^ In contrast, reductive agents like Na_2_S_2_O_4_ reduce chromophore groups within the dye molecules.^10^ Two-step NaOH/Na_2_S_2_O_4_ processes have demonstrated effective removal of reactive dye from cotton without severe cellulose degradation.^11^ Another two-step Na_2_S_2_O_4_/H_2_O_2_ decolorization process achieved >99.3% color removal while retaining >86% tensile strength and >96% fabric weight.^12^ A 3-step sequential decolorization process (acid/alkali → oxidation → reduction) has been carried out for reactive dyed cotton fabrics using either conventional heating or microwave assistance.^6^ Optimized conditions achieved decolorization efficiencies of 95–97% for the conventional method and 96–97% for the microwave-assisted method. However, the study evaluated only one dye (Reactive Yellow C4GL) on cellulosic fabric, which makes it difficult to determine whether the sequences are applicable across the textile industry. Another study reports that an alkali-reductive decolorization process effectively removes reactive dyes from cotton.^13^ Some studies show that two-step alkaline and reductive treatments can strip color from reactive dyed cotton.^8,14–19^ However, most of the studies focused only on one type of reactive dye and used high chemical dosage. Additionally, the multiple steps involved in color removal processes result in increased energy consumption, freshwater usage, and process complexity. Thus, it is important to find a viable strategy for the removal of persistent dyes from cotton waste.

There is limited information published in the literature on how one-bath and two-bath decolorization routes compare in terms of color removal performance, resource use, and fiber quality for industrially available mixed colored reactive-dyed cotton waste. Besides, to the best of our knowledge, the combined use of sodium carbonate and sodium dithionite for decolorization of reactive-dyed cotton textiles has not been previously reported. Therefore, the present study investigates and compares one-bath and two-bath alkaline-reductive decolorization processes for reactive-dyed cotton waste using Na_2_CO_3_ and Na_2_S_2_O_4_. The one-bath process integrates Na_2_CO_3_ and Na_2_S_2_O_4_ without discarding wastewater, whereas the two-bath process separates the two stages through intermediate washing, soaping, and drying. The main objective is to evaluate process efficiency and sustainability in terms of dye removal performance and potential contribution toward increasing the availability of clean, safe, and recyclable cellulosic materials.

## 2 Experimental

### 2.1 Materials

Reactive-dyed 100% cotton fabrics were obtained from Elyaf Tekstil (Bursa, Turkey). The fabrics were plain-woven from ring yarns with a fineness of 80/1 nm (number metric, m g^−1^) in both warp and weft directions. The warp and weft densities were 62.3 and 55 yarns per cm, respectively, and the fabric areal density was 94 g m^−2^. The dye characteristics of the reactive-dyed cotton fabrics are presented in Table 1. The reactive dyes in Table 1 are commercial, water-soluble dyes, and the fabrics were industrially dyed. Dye solubility was therefore not measured separately. Chemical structures of the reactive dyes used in the black, grey, navy, and dark green cotton fabrics are shown in Fig. 1. The decolorizing agents, such as sodium carbonate (BioXtra, ≥99.0%) and sodium dithionite (≥82%), were purchased from Sigma-Aldrich and used without further purification. The decolorizing agents (sodium carbonate and sodium dithionite) were selected based on the safe and sustainable by design (SSbD) framework and both are agents classified as level 2 out of level 3. The standard soap without optical brightening agent (SDC Reference Detergent Type 1) used for washing was purchased from SDC Enterprises Ltd (Thongsbridge, UK).

**Table 1:** Dye characteristics of the reactive-dyed 100% cotton fabrics [VS = vinylsulfone, MCT = monochlorotriazine]

Dyed fabric	Reactive dyes	Dye concentration (%)	Reactive groups	Total shade (%)
Black	Reactive Black 5	2.500	VS	7.500
	Reactive Orange 16			
	Reactive Orange 107			
	Reactive Black MIX	5.000	MCT	
Navy	Kimozol Extra Navy D	2.155	MCT & VS	4.530
	Reactive Red 195	1.045		
	Reactive Yellow 145	1.330		
Dark green	Reactive Red 195	0.253	MCT & VS	3.628
	Reactive Blue 222	2.000		
	Reactive Yellow 160	1.375		
Grey	Reactive Yellow 145	0.590	MCT & VS	2.280
	Reactive Blue 221	1.070		
	Reactive Red 195	0.620		

**Fig. 1:**
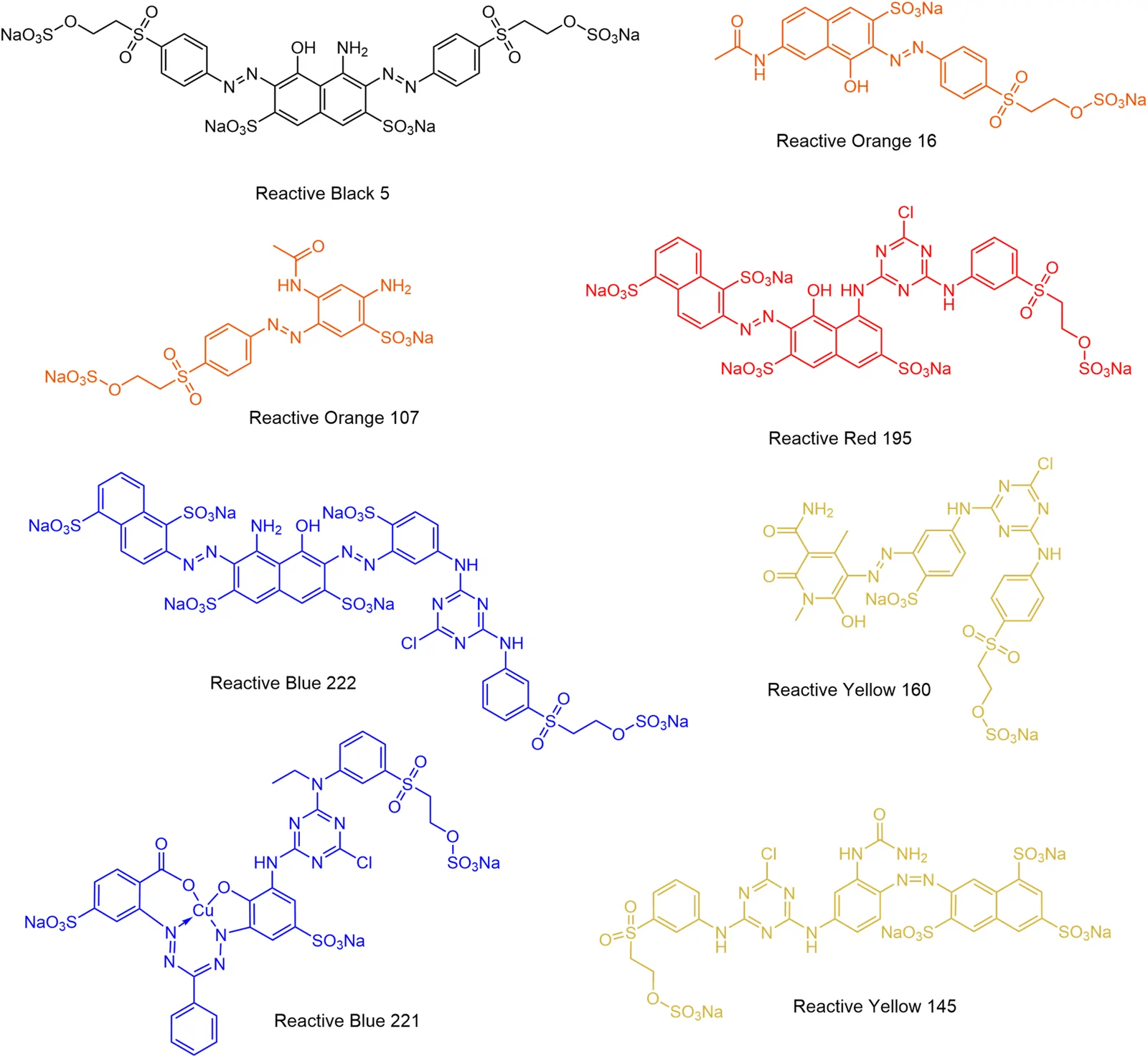
Chemical structures of the reactive dyes used in the black, grey, navy, and dark green cotton fabrics.

### 2.2 Decolorization process

#### 2.2.1 Sequential two-bath decolorization process

The two-bath decolorization of reactive-dyed cotton fabrics was carried out sequentially using sodium carbonate (Na_2_CO_3_) and sodium dithionite (Na_2_S_2_O_4_). In the first step, the fabrics were subjected to alkaline hydrolysis under defined processing conditions. After completion of this stage, the samples were thoroughly washed and rinsed. Subsequently, a second decolorization step was performed in a separate bath using sodium dithionite as the reducing agent. The fabrics were treated under comparable conditions, followed by thorough washing and drying (Fig. 2a).

**Fig. 2:**
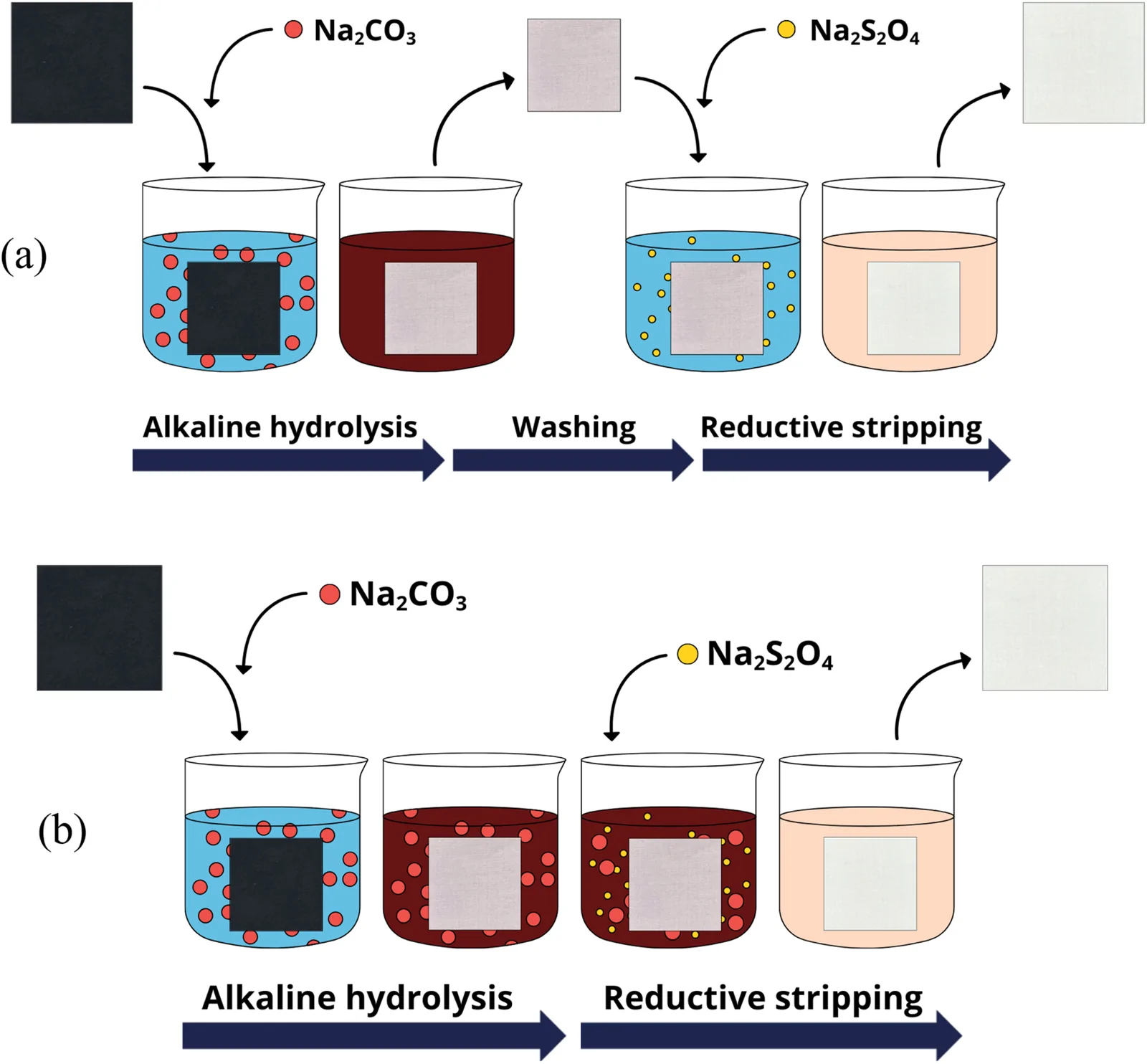
Schematic representation of the (a) sequential two-bath and (b) integrated one-bath decolorization processes using Na_2_CO_3_ and Na_2_S_2_O_4_. The high-temperature alkaline hydrolysis stage is followed by reductive stripping at a lower temperature.

#### 2.2.2 One-bath decolorization process

A one-bath decolorization process incorporating sodium carbonate and sodium dithionite was developed (Fig. 2b). As sodium carbonate decolorization produces an alkaline filtrate, sodium dithionite was subsequently introduced into the same bath to assess the influence of pH on its reducing performance. Instead of discarding the filtrate after Na_2_CO_3_ treatment, it was reutilized as the decolorization medium for the Na_2_S_2_O_4_ stage. This integrated approach enables both decolorization steps to be conducted within a single bath, thereby reducing process complexity and resource consumption. After the bath reached the target temperature, sodium dithionite was added directly, and the decolorization treatment was continued.

### 2.3 Performance evaluation

#### 2.3.1 Measurement of colorimetric properties

A reflectance spectrophotometer (X-Rite Ci7600 Spectrophotometer) was used to determine the CIE L*a*b* properties and color strength (*K*/*S*) of the fabrics. The measurements were repeated on each twice-folded sample at 10 various parts of the fabric to reduce errors caused by light reflection. The decolorization percentage was calculated using the *K*/*S* values for both original dyed fabrics and decolorized fabrics using eqn (1). In the equation, (*K*/*S*)_0_ represents the relative color strength of the original dyed fabric, and (*K*/*S*)_f_ represents the relative color strength of the decolorized sample.1
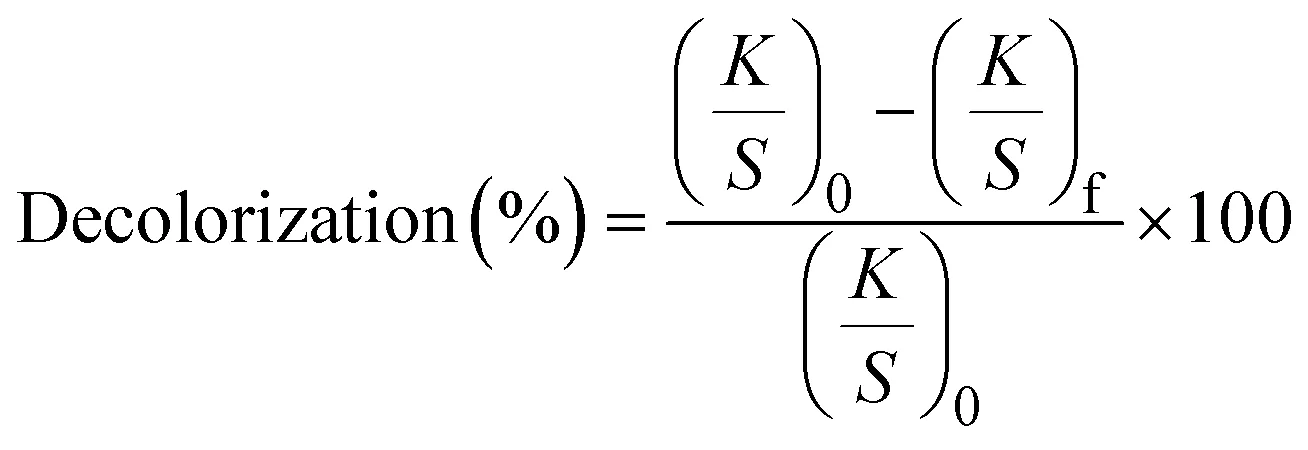


Although the fabrics can obtain high lightness and decolorization percentage after decolorization treatments, they may still retain noticeable color. Therefore, the whiteness index (WI) was calculated to evaluate the whiteness of the fabrics after each treatment (eqn (2)).^8^2
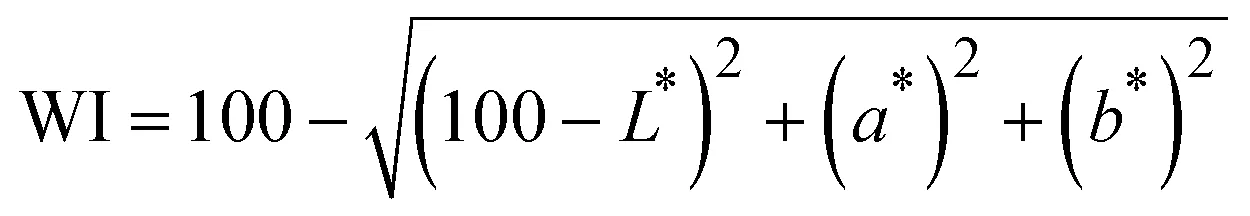
where the L*, a*, and b* are the lightness, green-red, and blue-yellow values, respectively. A whiteness index value above 80 was targeted because it is generally considered acceptable in the textile industry.^8^

#### 2.3.2 Physical and mechanical properties

Tensile strength tests were conducted in accordance with SFS-EN ISO 13934-1:2013, with minor modifications to specimen dimensions, using a universal testing machine (Instron 4204). A 5 kN load cell equipped with screw-action tensile grips was employed, with a gauge length of 100 mm and an extension rate of 50 mm min^−1^. Specimens measuring 25 × 150 mm (excluding fringes) were prepared with sufficient width to prevent yarn slippage. Prior to testing, all samples were conditioned for 48–72 h under standard atmospheric conditions (20 °C, 65% relative humidity) in accordance with ISO 139:2005. Tensile properties were measured in both warp and weft directions, with 5–8 replicates performed to ensure data reliability. The tensile strength retention was calculated using eqn (3).3
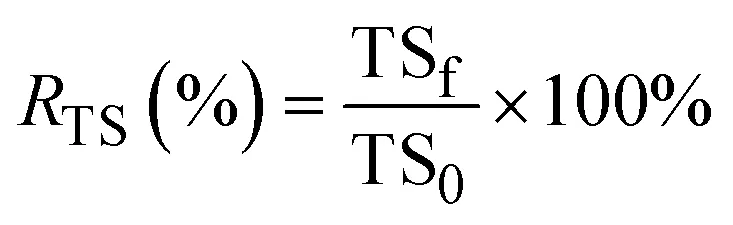
where TS_f_ is the tensile strength of the fabric after decolorization, and TS_0_ is the tensile strength of the original dyed fabric.

The weight loss percentage of the fabrics after treatment was analyzed using eqn (4). Before measuring weight, the fabrics were conditioned for 72 hours.4
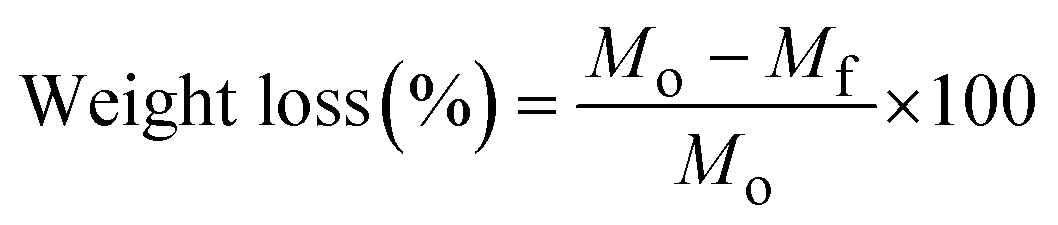
where, *M*_o_ = fabric weight before decolorization and *M*_f_ = fabric weight after decolorization.

#### 2.3.3 Measurement of intrinsic viscosity and degree of polymerization

The intrinsic viscosity and degree of polymerization were determined according to the SCAN-CM 15:88 standard method. Approximately 150 mg of ground cotton was dissolved in Milli-Q water and cupriethylenediamine (CED) solution using a shaker. The intrinsic viscosity of the resulting solutions was measured with a capillary viscometer. The relationship between intrinsic viscosity (*η*) and degree of polymerization (DP) is described by the rearranged Mark–Houwink equation (eqn (5)).^20^5
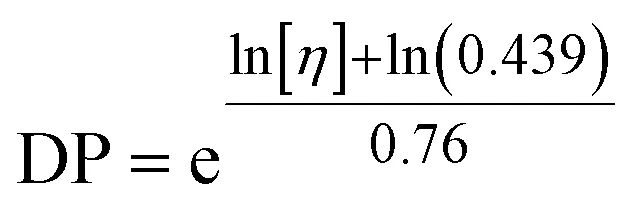


#### 2.3.4 Elemental analysis

The CHNS analysis was performed with Thermo Scientific FlashSmart EA CHNS/O with MV (Thermo Fisher Scientific, Waltham, MA, USA). Triplicates of each sample were measured, and their average was reported. Sulfanilamide was used as a standard. The elemental composition of the sulfanilamide standard was 16.31% nitrogen, 41.79% carbon, 4.65% hydrogen, and 18.62% sulfur. The CHNS analysis was performed under the following conditions: furnace temperature of 950 °C, carrier gas flow rate of 100 mL min^−1^, oxygen flow rate of 250 mL min^−1^, 5 min injection time, and oven temperature of 65 °C.

#### 2.3.5 X-ray diffraction analysis (XRD)

The crystallinity was analyzed using a Xenocs Xeuss 3.0 diffractometer, equipped with a 2D Dectris Eiger2 R 1M detector positioned 56 mm from the specimen. Measurements were performed in transmission mode using Cu Kα radiation (*λ* = 1.54189 Å) at an operating voltage of 50 kV and current of 0.6 mA. The scattering profiles were corrected using data from a blank run. Following the established procedure, the crystallinity index (CI) was calculated by the well-established Segal method, comparing the total intensity of (200) and the amorphous intensity at 18° 2*θ*.^21^ Additionally, the crystallinity was analyzed *via* the use of a Savitzky–Golay filter to smooth the data with a window size of 29 (polynomial order of 1) and the determination of the background profile by means of a window of 201. The ratio of the total scattered intensity area *vs.* background intensity was formed, as described in eqn (6)^22^.6
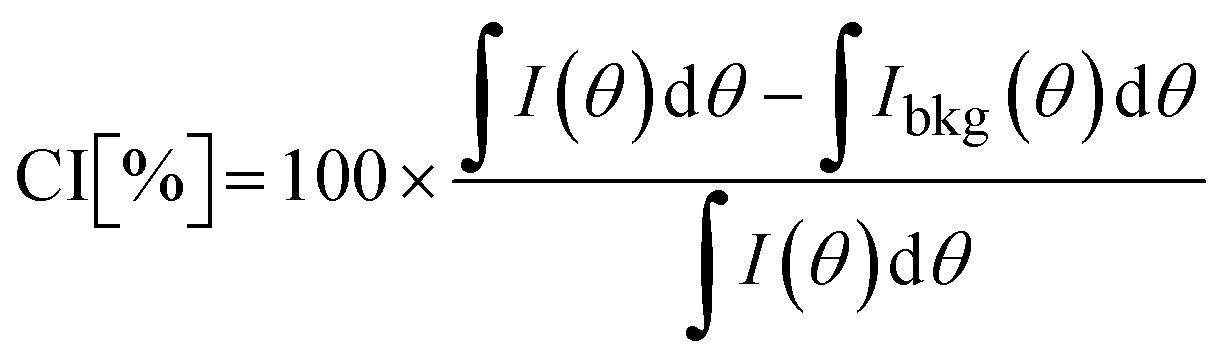


#### 2.3.6 Scanning electron microscopy

The scanning electron microscopy (SEM, Zeiss Sigma VP) was used to evaluate the effect of decolorization treatment on the fabric surface. Prior to imaging, a thin layer (approximately 8 nm) of gold–palladium alloy was deposited onto the samples using a Leica EM high-vacuum sputter coater. SEM images were then captured at an accelerating voltage of 3 kV. Energy-dispersive X-ray (EDX) analysis was also performed using a scanning electron microscope (SEM) to identify the elemental composition.

#### 2.3.7 Thermogravimetric analysis

Thermogravimetric analysis (TGA) of the fabric samples was performed using a TGA 5500 instrument under a nitrogen atmosphere with a flow rate of 25 mL min^−1^. Approximately 5–10 mg of each sample was placed on a platinum pan and introduced into the heating furnace. The analysis was conducted over a temperature range of 30 to 600 °C at a heating rate of 10 °C min^−1^.

#### 2.3.8 X-ray photoelectron spectroscopy analysis

X-ray photoelectron spectroscopy (XPS) measurements were performed using a Kratos AXIS Ultra DLD spectrometer equipped with a monochromated Al Kα X-ray source (1486.7 eV, 100 W). Both survey and high-resolution spectra were acquired under ultrahigh-vacuum conditions (<1 × 10^−9^ Torr) using pass energies of 80 and 20 eV, respectively. To assess surface homogeneity and ensure representative measurements, spectra were collected from three different locations on each sample.^23^

All fabrics were conditioned in an air-conditioned room prior to characterization at a temperature of 23 ± 2 °C and a relative humidity of 65 ± 1%.

## 3 Results and discussion

### 3.1 Process parameters optimization

The decolorization performance was evaluated based on lightness (L*), with a target of achieving complete decolorization (L* > 70) while preserving fiber integrity. The effects of sodium carbonate concentration, temperature, and treatment time were systematically investigated.

Increasing the Na_2_CO_3_ concentration from 5 to 30 g L^−1^ resulted in a progressive improvement in decolorization (Fig. 3A). At 10 g L^−1^, the L* value reached approximately 70, indicating effective color removal. Although higher concentrations further increased L*, they also caused noticeable fabric degradation. Therefore, 10 g L^−1^ was selected as the optimal concentration, providing a balance between decolorization efficiency and fabric preservation. The treatment temperature had a pronounced effect on performance (Fig. 3B). At 120 °C, insufficient decolorization was observed (L* ≈ 60), whereas increasing the temperature to 140 °C resulted in higher lightness values (L* > 80) but caused severe material degradation. At 130 °C, effective decolorization (L* ≈ 70) was achieved without significant damage to the fabrics, and this temperature was therefore considered optimal.

**Fig. 3:**
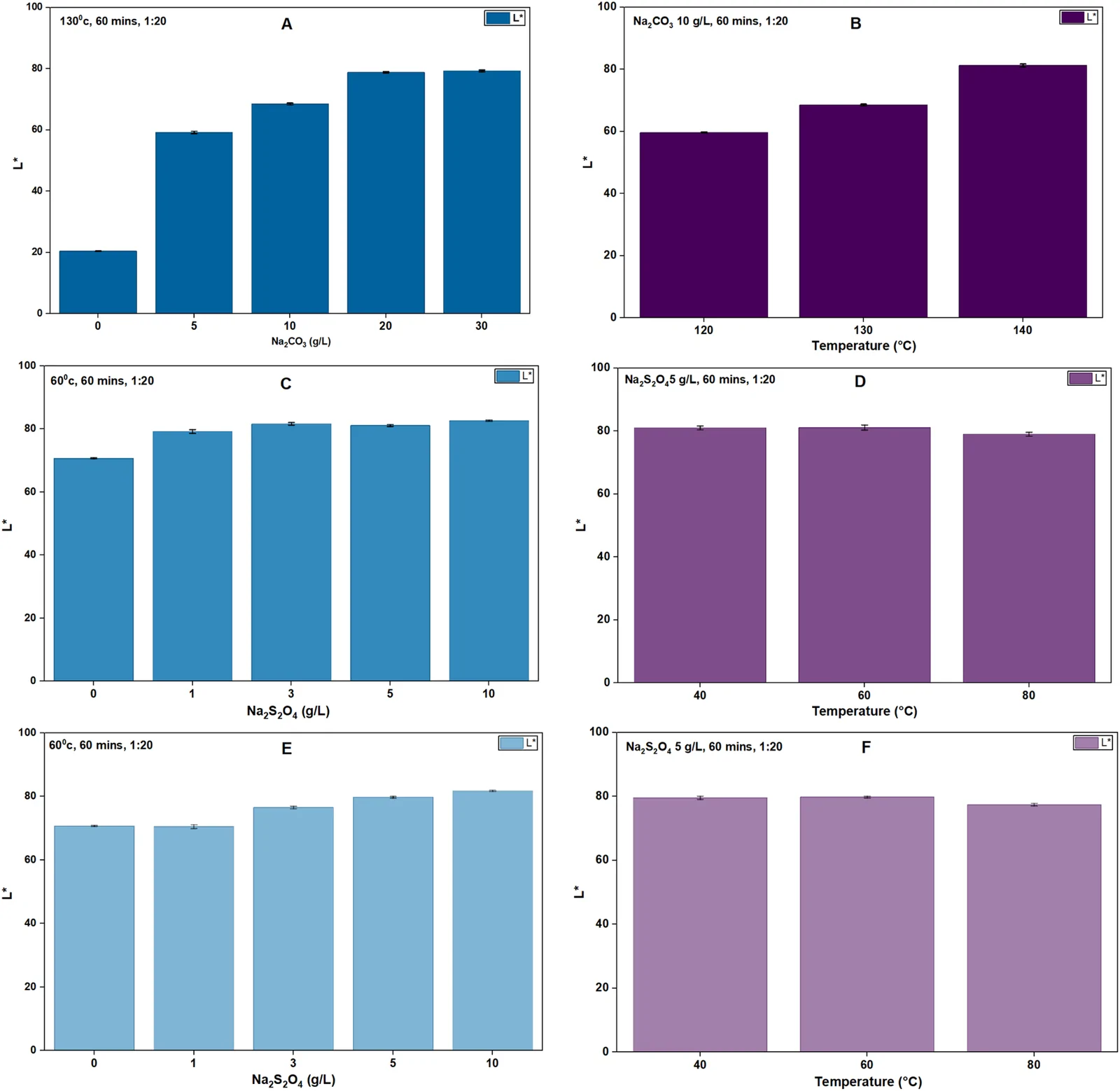
Optimization of process parameters for decolorization: (A and B) concentration and temperature effects during alkaline hydrolysis; (C and D) optimization of reductive treatment with sodium dithionite in the two-bath process; and (E and F) optimization of reductive treatment in the one-bath process, where sodium dithionite is added directly to the alkaline bath after temperature reduction (Iindicate the standard error).

Similarly, treatment time influenced the outcome. A duration of 60 min provided sufficient decolorization, while shorter times led to incomplete color removal, and longer treatments did not yield significant additional benefits but increased the risk of fabric degradation (Fig. S1).

The optimization of processing parameters for both two-bath and one-bath reductive decolorization processes (Fig. 3C–F) followed the same protocol as alkaline hydrolysis. In both cases, an increase in the concentration of decolorizing chemicals improved decolorization uniformity and enhanced the L* value marginally. Subsequently, temperature optimization highlighted the need for a moderate temperature of 60 °C for effective and uniform color removal in the reduction stage, both for the two-step and one-step approaches. Further increase in temperature led to a slight decrease in the L* value, while a decrease in temperature led to uneven decolorization. In addition, cooling the treatment bath from 130 to below 60 °C in the one-bath process requires considerable time; therefore, further lowering the temperature was considered impractical. Similarly, treatment duration was optimized at 60 min, as an increase in time did not have any significant improvement in the L* value (See Fig. S1). While shorter treatment times were also efficient, they are not feasible on an industrial scale since longer process times are often needed for sufficient chemical penetration. Although the two-bath results indicate fast and efficient decolorization even at room temperature and low concentrations, the resulting fabric is unevenly decolorized. The results indicate that Na_2_S_2_O_4_ reacts fast with dye molecules, regardless of the temperature, yet it requires time and moderate heat to uniformly penetrate and decolorize the fabric. The optimal conditions for these decolorization processes were determined by achieving satisfactory decolorization performance while maintaining fabric integrity. The optimized parameters for each process are presented in Table 2.

**Table 2:** The optimized process parameters for the alkaline hydrolysis (Na_2_CO_3_), two-bath (Na_2_CO_3_/Na_2_S_2_O_4_), and one-bath (Na_2_CO_3_/Na_2_S_2_O_4_) treatments

Process step	Chemical	Concentration (g L^−1^)	Temperature (°C)	Time (min)	M : L
1st step: alkaline hydrolysis	Na_2_CO_3_	10	130	60	1 : 20
2nd step: two-bath process	Na_2_S_2_O_4_	5	60	60	1 : 20
2nd step: one-bath process	Na_2_S_2_O_4_	5	60	60	1 : 20

### 3.2 Color measurement properties

The goal of decolorization was to achieve ≥99% decolorization with a lightness (L*) value >80, supporting their potential as a high-quality feedstock for textile circularity. The colorimetric data is presented in Table S1. Fig. 4(a) and (b) present the decolorization percentage and lightness reached on the fabrics after each decolorization treatment, respectively. After alkaline hydrolysis, the black and navy fabrics exhibited decolorization exceeding 99%, while grey and dark green fabrics showed significantly lower values. This can be due to black fabric being dyed with monofunctional and homo-bifunctional reactive dyes, whereas the other fabrics were dyed with hetero-bifunctional dyes containing both vinyl sulfone and monochlorotriazine reactive groups. The presence of two reactive groups makes the dyes more resistant to decolorization because two different reactive groups have different chemical reactivities under different conditions and are crosslinked better with the cellulose chain. The high decolorization percentage indicates that significant dye removal occurred due to fiber swelling and the cleavage of dye–fiber bonds under alkaline conditions at elevated temperature.^24^ As the decolorization percentage is calculated from *K*/*S* value of the fabric, black and navy fabrics have relatively high decolorization percentages due to their dark shade.

**Fig. 4:**
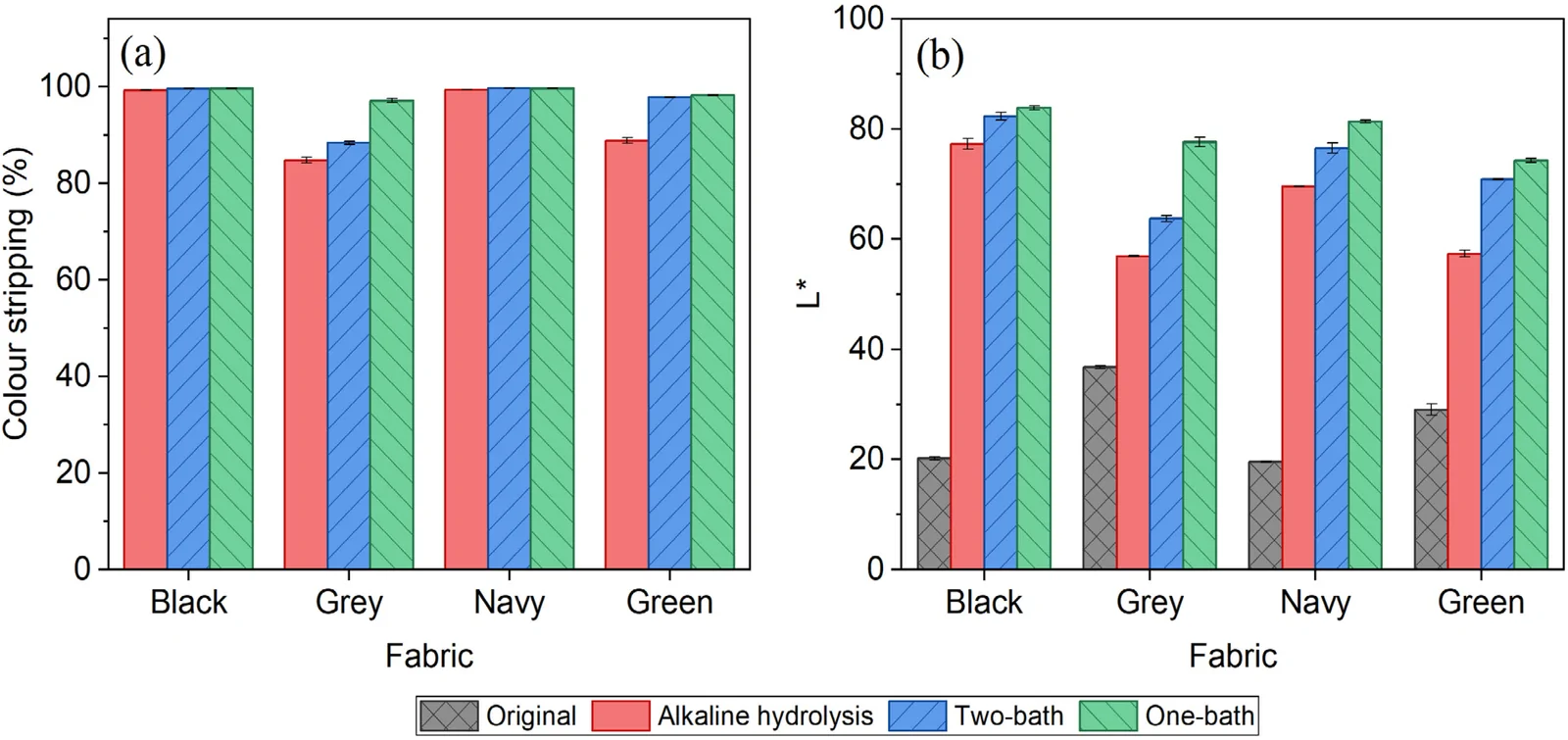
(a) Decolorization (%) and (b) the lightness (L*) of four different fabrics before and after each decolorization treatment using optimized recipes.

Additionally, the one-bath and two-bath processes provided clearly different decolorization results. Although the grey fabric contained the lowest overall dye concentration, the decolorization results indicated that it was the most difficult to treat. While other fabrics reached above 97% decolorization with both two- and one-bath methods, the grey fabric exhibited much lower decolorization percentages. This behavior can be attributed to the chemical nature of the dyes present (See Fig. 1), particularly the high proportion of Reactive Blue 221 (RB221), which was only partially removed or chemically modified during the decolorization process. RB221 is a copper-complex dye containing a coordinated Cu^2+^ ion, which enhances its stability and affinity for the fiber.^25,26^ Under alkaline and reductive conditions, complete removal of such metal-complex dyes is more challenging, as the chromophore system and metal coordination can remain partially intact. In addition, the copper species may possibly persist on the fabric surface in the form of insoluble Cu(ii) compounds, contributing marginally to the residual blue coloration.^27^ The XPS results confirmed the presence of copper, with a Cu 2p atomic concentration of 0.17 ± 0.04% (see Table S2). In addition, SEM-EDX also detected Cu in the grey fabric and results are presented in Fig. 5. These results provide analytical evidence supporting the presence of a copper-containing dye species in the grey fabric. In contrast, no Cu was identified in the undyed cotton fabric.^28^

**Fig. 5:**
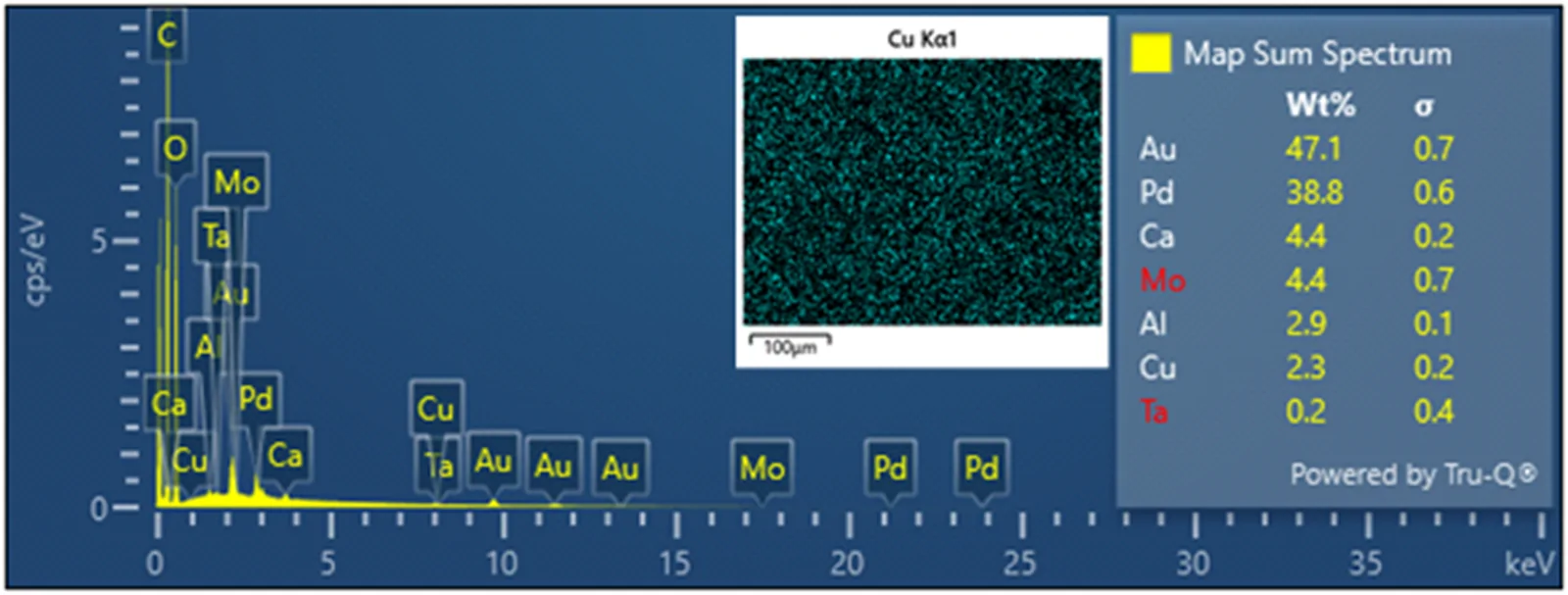
Traces of copper (Cu) from energy-dispersive X-ray spectroscopy after decolorization with Na_2_CO_3_–Na_2_S_2_O_4_.

Across all fabrics, the lightness reached or came close to the target lightness of 80 after the one-bath process, as shown in Fig. 4(b). The two-bath process, on the other hand, was not effective enough to reach such high lightness values in fabrics other than the black fabric. One possible explanation is that, in the one-bath process, Na_2_S_2_O_4_ is introduced into an alkaline medium that is close to the optimal pH for decolorization. In contrast, in the two-bath process, the pH of the reductive stage was not adjusted. If a small amount of sodium carbonate had been added to the second bath to control the pH, comparable performance might have been achieved. The lightness and whiteness index results are quite similar, but the whiteness index provides more information about the degree of fabric decolorization. In all cases, the whiteness index was slightly lower than the lightness of the fabric (See Fig. S2). However, the navy fabric showed considerably lower WI values for the decolorized fabrics when compared to their lightness. This can be explained by visually examining the decolorization of navy fabric; it is observed that the alkaline hydrolysis treatment successfully removed the navy color, producing fabric with a pink hue. This suggests that most of the Kimozol Extra Navy D dye was removed during alkaline hydrolysis, leaving only Reactive Red 195 and Reactive Yellow 145 bonded with cotton fabric. The residual pink coloration was further reduced by the two-bath process and was no longer visible after the one-bath treatment, which produced a light-yellow fabric. Thus, while the achieved lightness was high, the WI value was lower due to the distinct pink or yellow colorations following decolorization. The observed residual yellow shade may also be a result of Reactive Red 195 partially degrading into yellow-colored compounds during the reductive treatment of the one-bath process.^29^ Visual representations of the black, grey, navy, and dark green fabrics before and after the decolorization treatments are presented in Table 3.

**Table 3:** Camera images of the black, grey, navy, and dark green fabrics before and after the decolorization treatments: alkaline hydrolysis (Na_2_CO_3_), two-bath (Na_2_CO_3_/Na_2_S_2_O_4_), and one-bath process (Na_2_CO_3_/Na_2_S_2_O_4_). The camera may make the colors appear slightly more intense than those of the actual samples. Therefore, the images are provided for visual comparison and may not exactly represent the samples' true colors

Fabric	Dyed	Alkaline hydrolysis^a^	Two-bath^a^^,^^b^	One-bath^a^^,^^b^	Undyed reference
Black	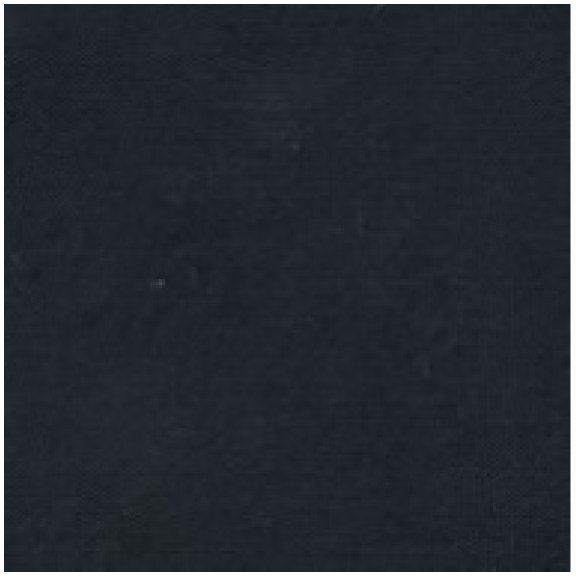	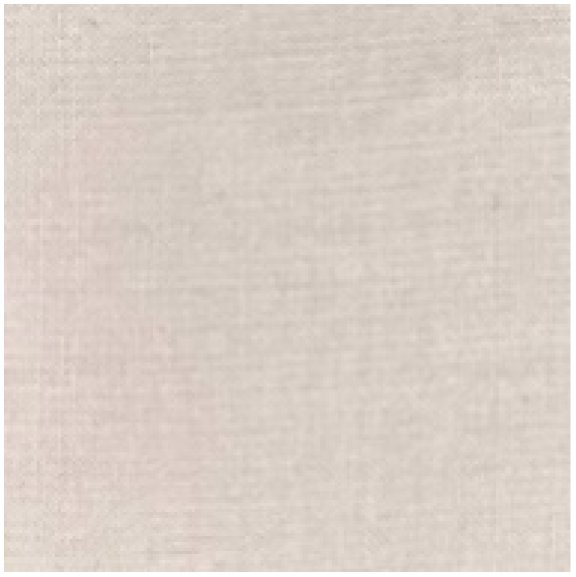	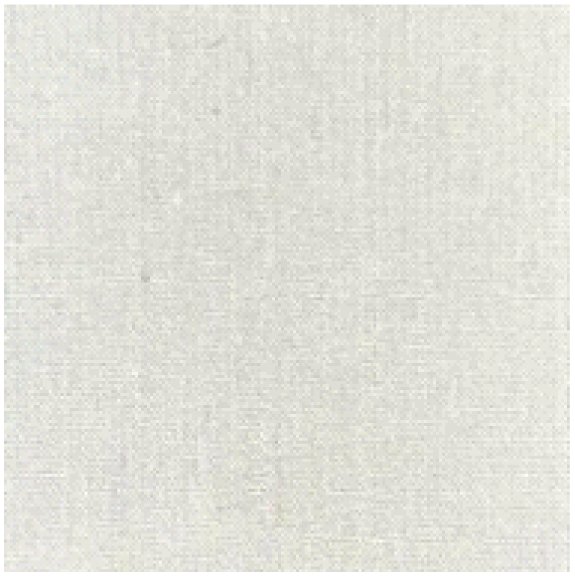	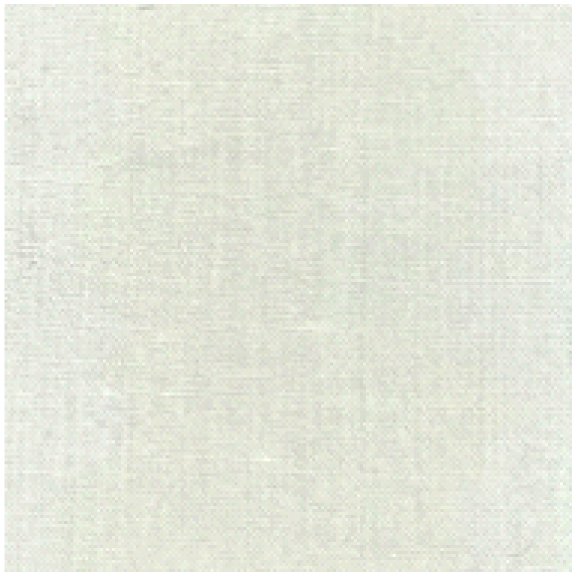	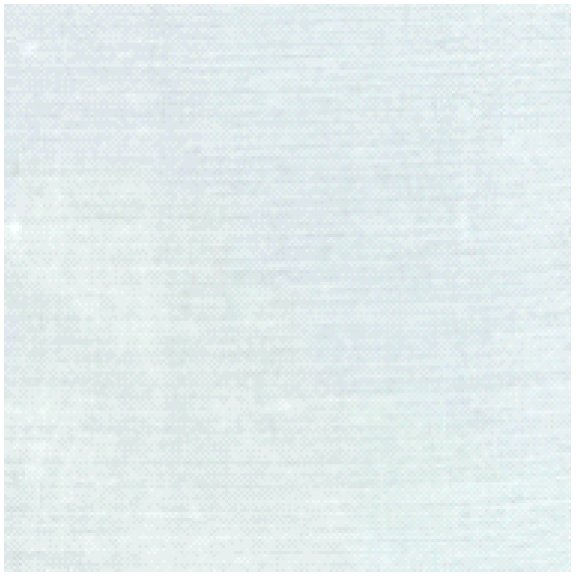
Grey	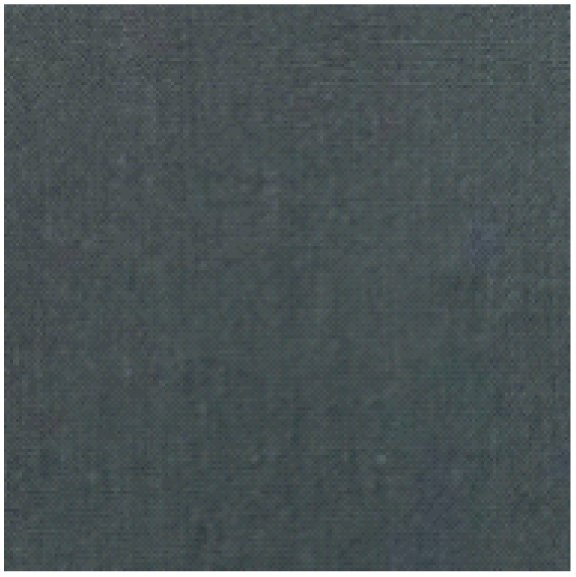	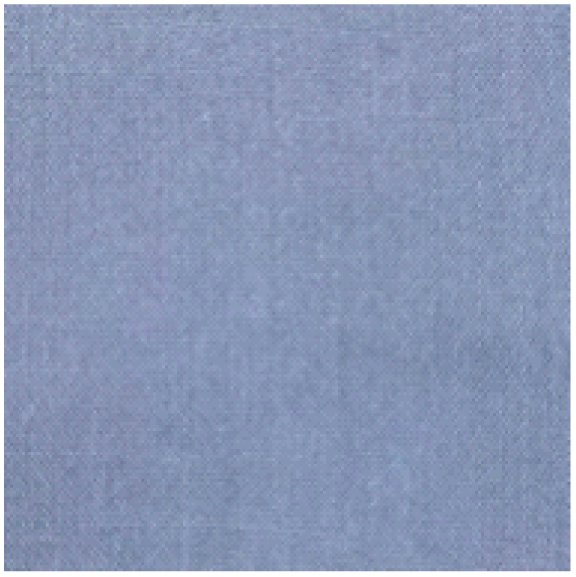	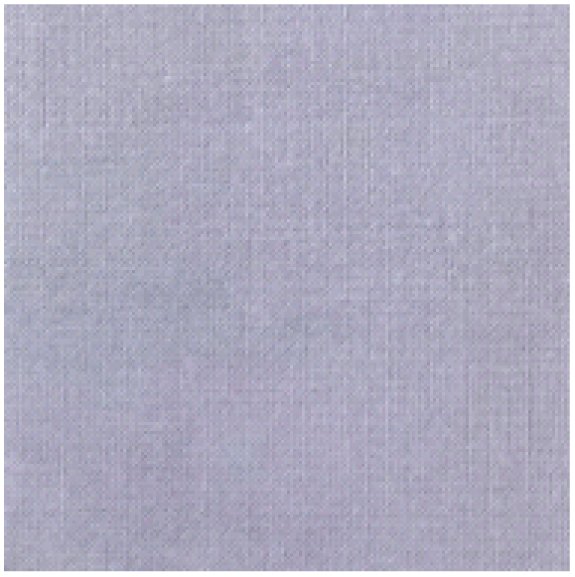	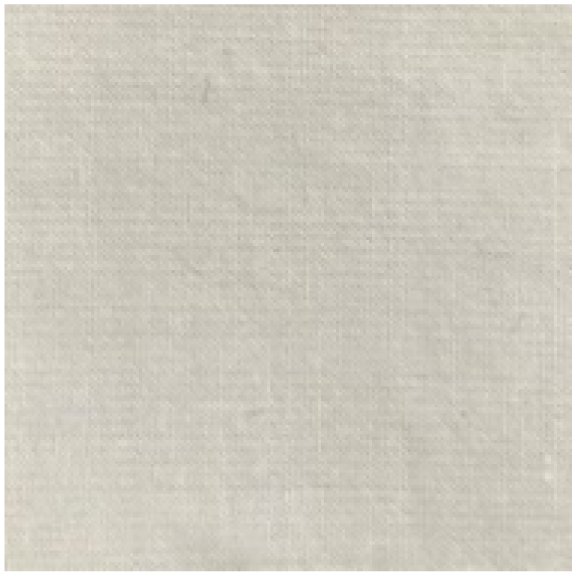	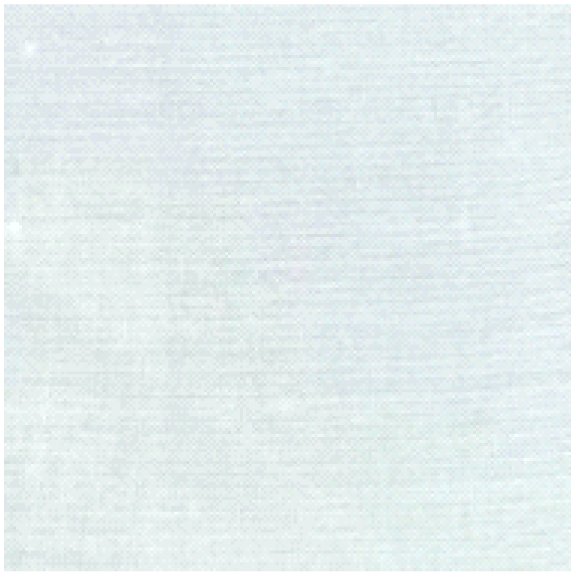
Navy	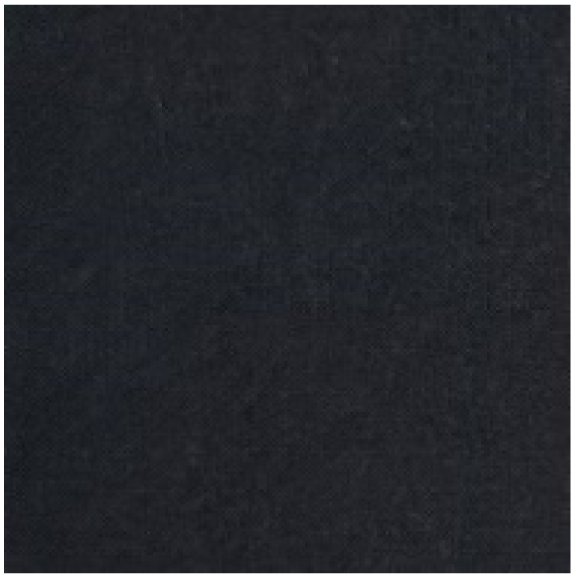	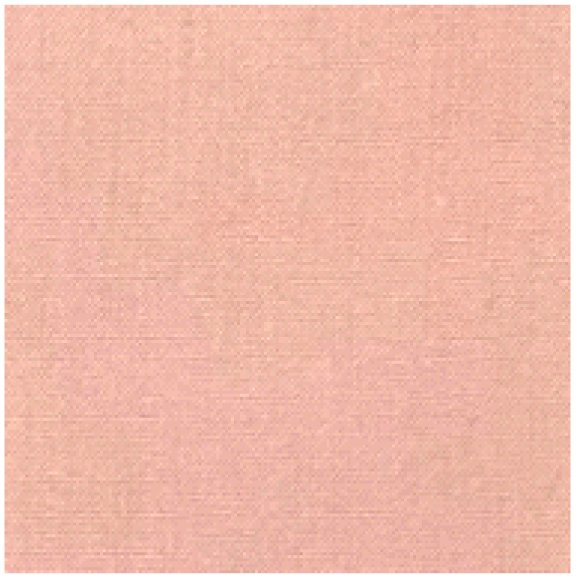	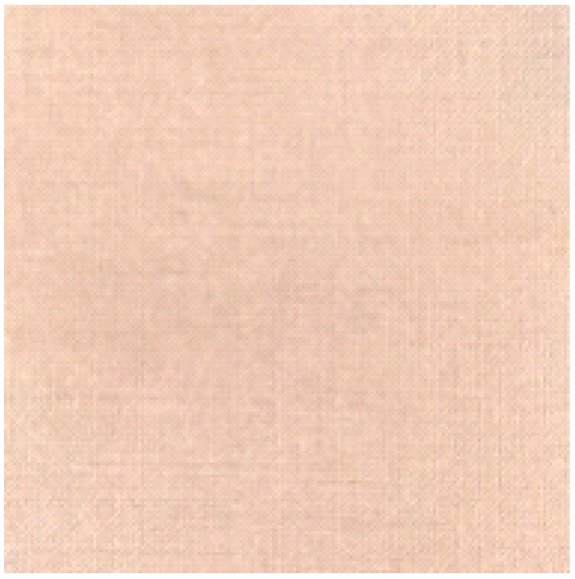	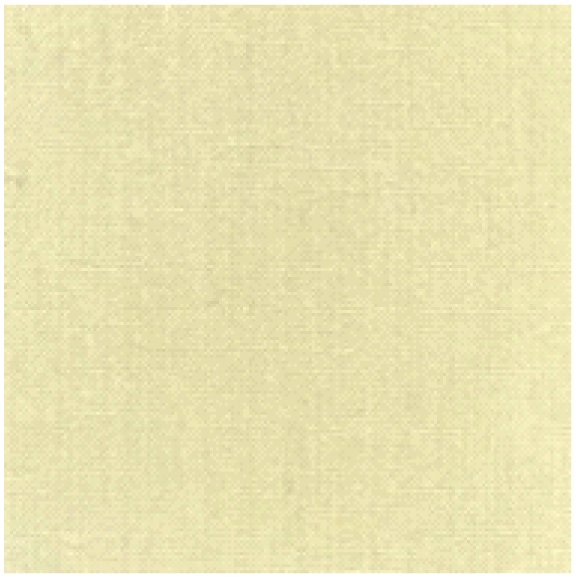	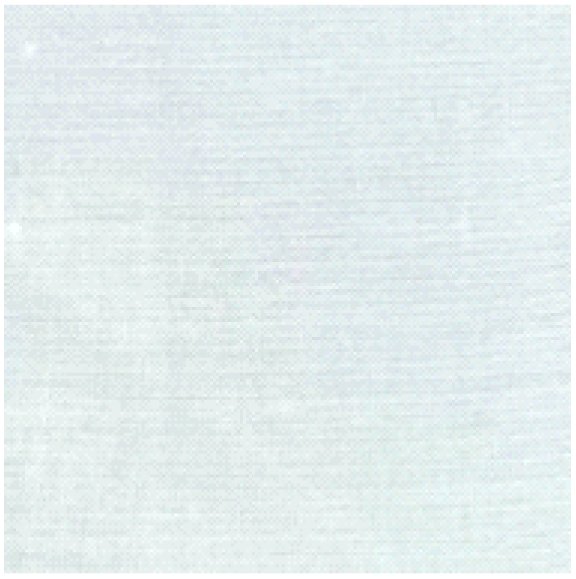
Dark green	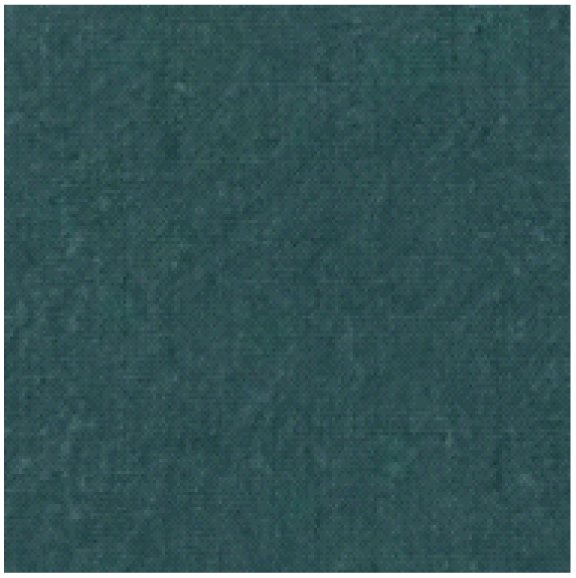	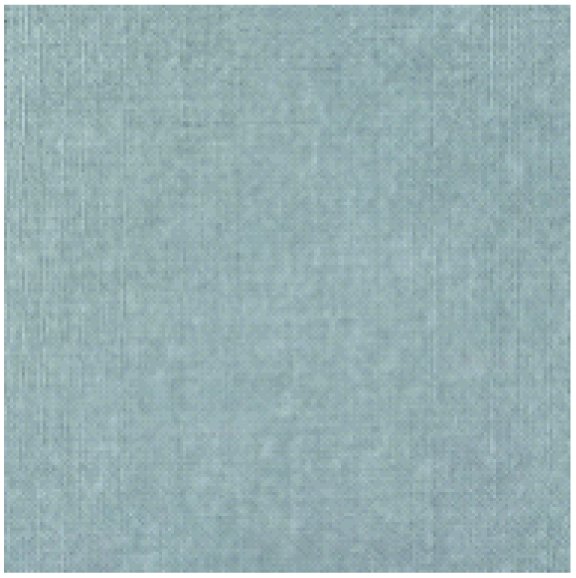	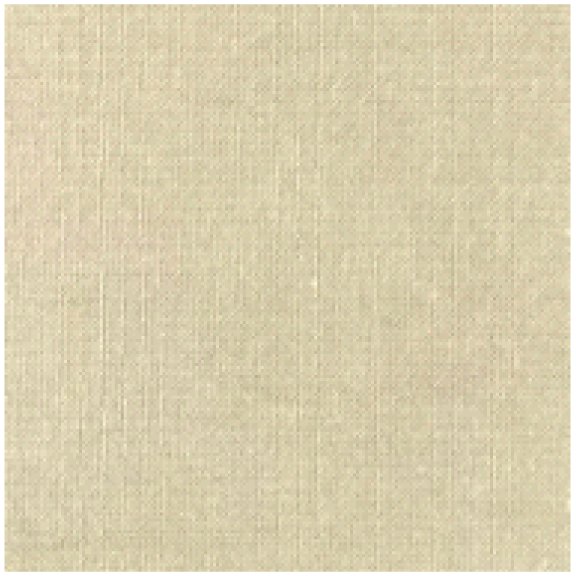	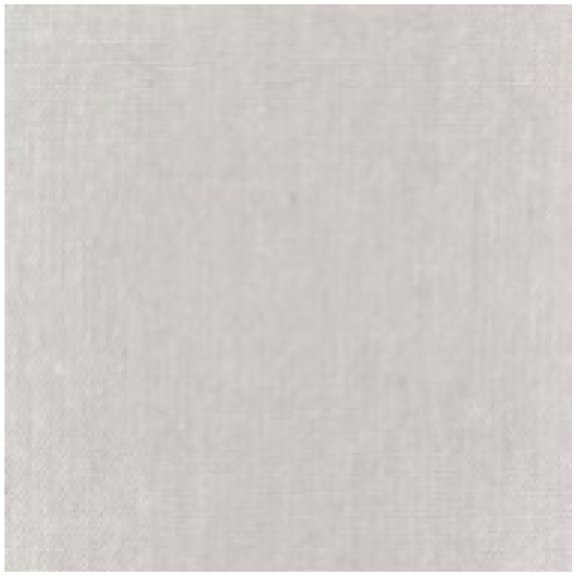	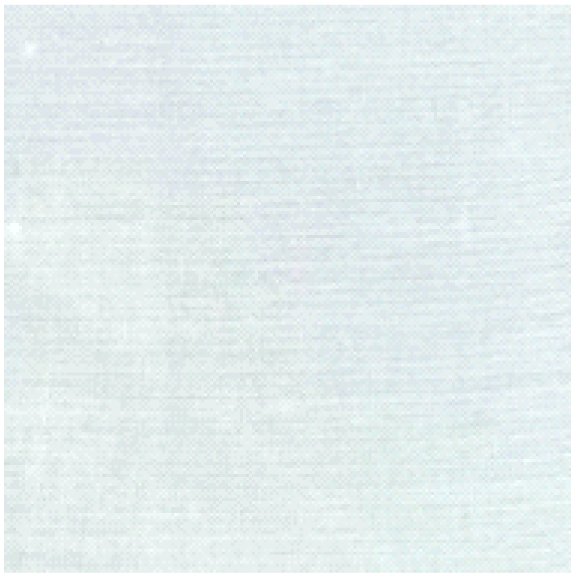

a 1st decolorization step: [Na_2_CO_3_] 10 g L^−1^, 130 °C, 60 min, 1 : 20.

b 2nd decolorization step: [Na_2_S_2_O_4_] 5 g L^−1^, 60 °C, 60 min, 1 : 20.

The changes in the *K*/*S* curves of the fabrics after each decolorization treatment support the interpretation of the colorimetric results (See Fig. S3). For the grey and dark green fabrics, the *K*/*S* curves show that the maximum absorption peaks have remained at the same wavelengths as before Na_2_CO_3_ decolorization, indicating that the treatment could not achieve complete color removal. In contrast, the black and navy fabrics exhibited different behavior, as the *K*/*S* values decreased significantly after Na_2_CO_3_ decolorization, and no significant peak was observed in the visible spectrum (360–750 nm). The color of the fabrics changed from black and navy to beige and pink, respectively, reflecting much more efficient dye removal. After the sequential treatments, the *K*/*S* curves further flattened, and the maximum absorption peaks disappeared. However, the *K*/*S* curves of the two-bath-decolorized grey and dark green fabrics still displayed absorption peaks like those before decolorization. This phenomenon is not observed with the one-bath-decolorized fabrics, which displayed flat *K*/*S* curves, indicating that the dye molecules were more effectively degraded by Na_2_S_2_O_4_ in the one-bath process.

### 3.3 Mechanical properties

As shown in Fig. 6, all decolorization methods resulted in measurable weight loss, strongly influenced by the shade percentage of the fabric and processing route. For instance, the highest weight loss was observed for the black fabric compared to the other colored fabrics. This can be attributed to its high shade depth (7.5% on the weight of the fabric). The colorimetric results indicate substantial color removal during the decolorization processes. The removal of dye-related species may have contributed to the observed weight loss, although losses of other fabric components cannot be excluded. A similar study has also reported the effect of shade percentage on the weight loss of the fabric, where they found more weight loss on the fabric for 5% shade than 2.5% shade under the same processing conditions.^17^ Again, the type of treatment had a considerable effect on weight loss properties. After alkaline hydrolysis, a weight loss of 7.9% was observed for the black fabric. However, the weight loss increase was minimal when these alkaline-treated fabrics were subjected to reductive treatment in a two-step process. An overall increase in the weight loss of black fabric was observed, from 7.9% to 8.1% for both two-bath and one-bath treatments. This is because most of the components, such as low-molecular-weight fragments and easily cleavable cellulose regions, were hydrolyzed during alkali treatment. On the other hand, reductive treatment selectively attacked chromophore groups, leaving the cellulose backbone chain unaffected.^16^ A similar trend was observed for green, grey, and navy fabrics as well. Grey fabric, with a shade percentage of 2.2%, suffered the least amount of weight loss amongst other fabrics.

**Fig. 6:**
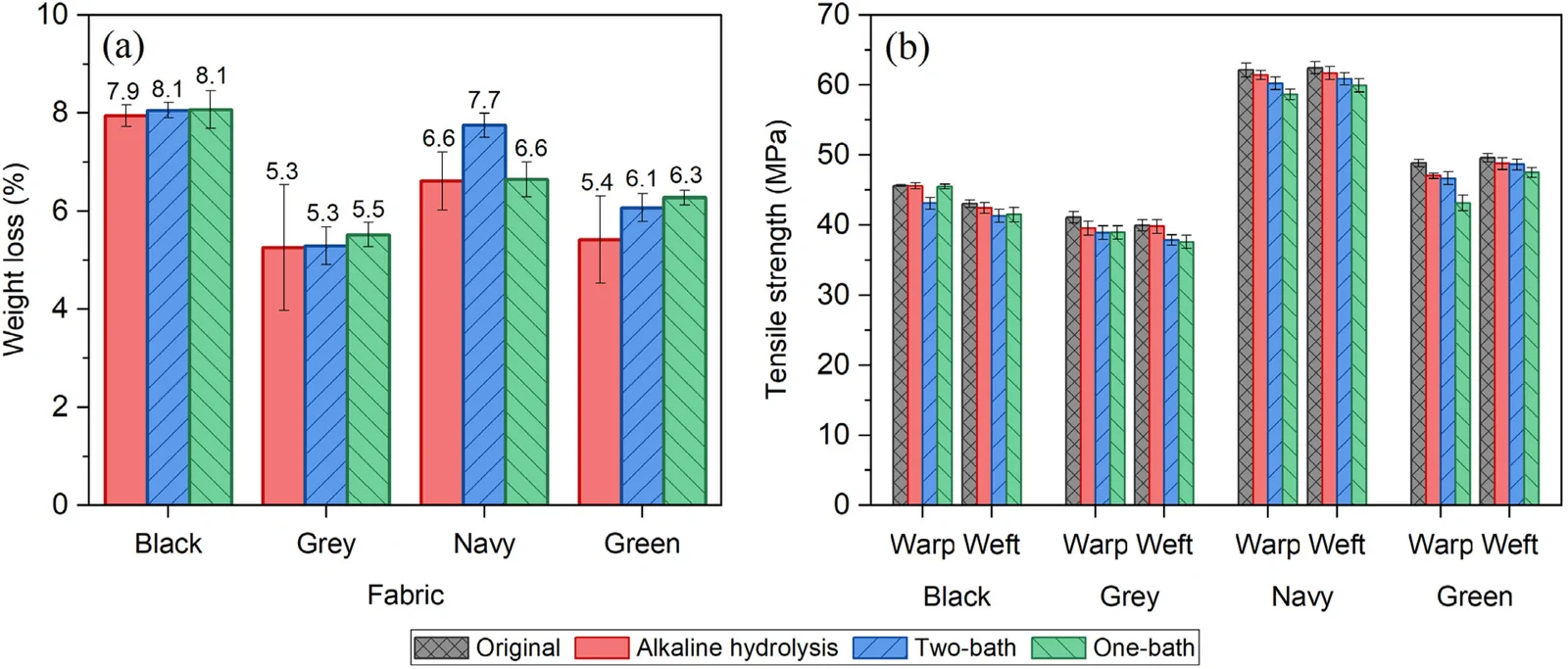
(a) Weight loss (%) and (b) tensile strength (MPa) of the treated fabrics after decolorization processes.

The tensile properties of the colored fabrics after each decolorization process were evaluated and compared with those of the dyed fabric, as shown in Fig. 6b. As can be seen in this figure, the tensile strength was retained well after all decolorization processes. Table S3 shows the tensile strength and calculated percentage tensile strength retention values compared to the original dyed fabric. After the sequential two-bath and one-bath treatments, the tensile strength retention was more than 94% for the black, grey, and navy fabrics. The green fabric exhibited similar strength retention, except for a more notable tensile strength decrease in the warp direction after the one-bath treatment. However, the tensile strength retention was above 88%, meaning that satisfactory tensile strength retention was achieved. Overall, the tensile strength retention was excellent across all decolorization sequences. A slight decrease in tensile strength of the decolorized fabric would indicate that the treatment had degraded cellulose chains and decreased their molecular weight.^11^ Unlike harsh oxidative treatments using, *e.g.*, hydrogen peroxide or sodium hypochlorite, the sequential alkaline hydrolysis-reductive decolorization treatment did not break the cellulose chain, leading to the preservation of the mechanical performance.^5,10^ Thus, the proposed Na_2_CO_3_/Na_2_S_2_O_4_ sequential treatments are suitable for mild decolorization of reactive-dyed cotton waste, resulting in good reusability of the treated fabric in both chemical and mechanical recycling processes.

### 3.4 Morphological analysis

SEM micrographs (Fig. 7) indicate that the reactive-dyed cotton fibers have a characteristic flat, ribbon-like morphology with natural convolutions, compact fibrillar packing, and a relatively smooth, intact surface, with minor dye deposits visible in Fig. 7A. After alkaline treatment with Na_2_CO_3_, increased surface roughness and slight micro fibrillation are observed due to partial disruption of the hydrogen-bonded cellulose network under alkaline conditions at elevated temperature (Fig. 7B);^30^ however, the spiral structure and parallel ridges remain visible, indicating preserved structural integrity.^31^ Following reductive treatment with Na_2_S_2_O_4,_ the fiber surface appears smoother with reduced micro fibrillation (Fig. 7C and D),^10^ as the reducing agent primarily affects dye chromophores rather than cellulose–dye covalent bonds, resulting in minimal fiber damage. No significant morphological differences are evident between one-bath and two-bath decolorization processes (Fig. 7C and D), confirming that the treatments cause only minor surface modifications without substantial fiber degradation.

**Fig. 7:**
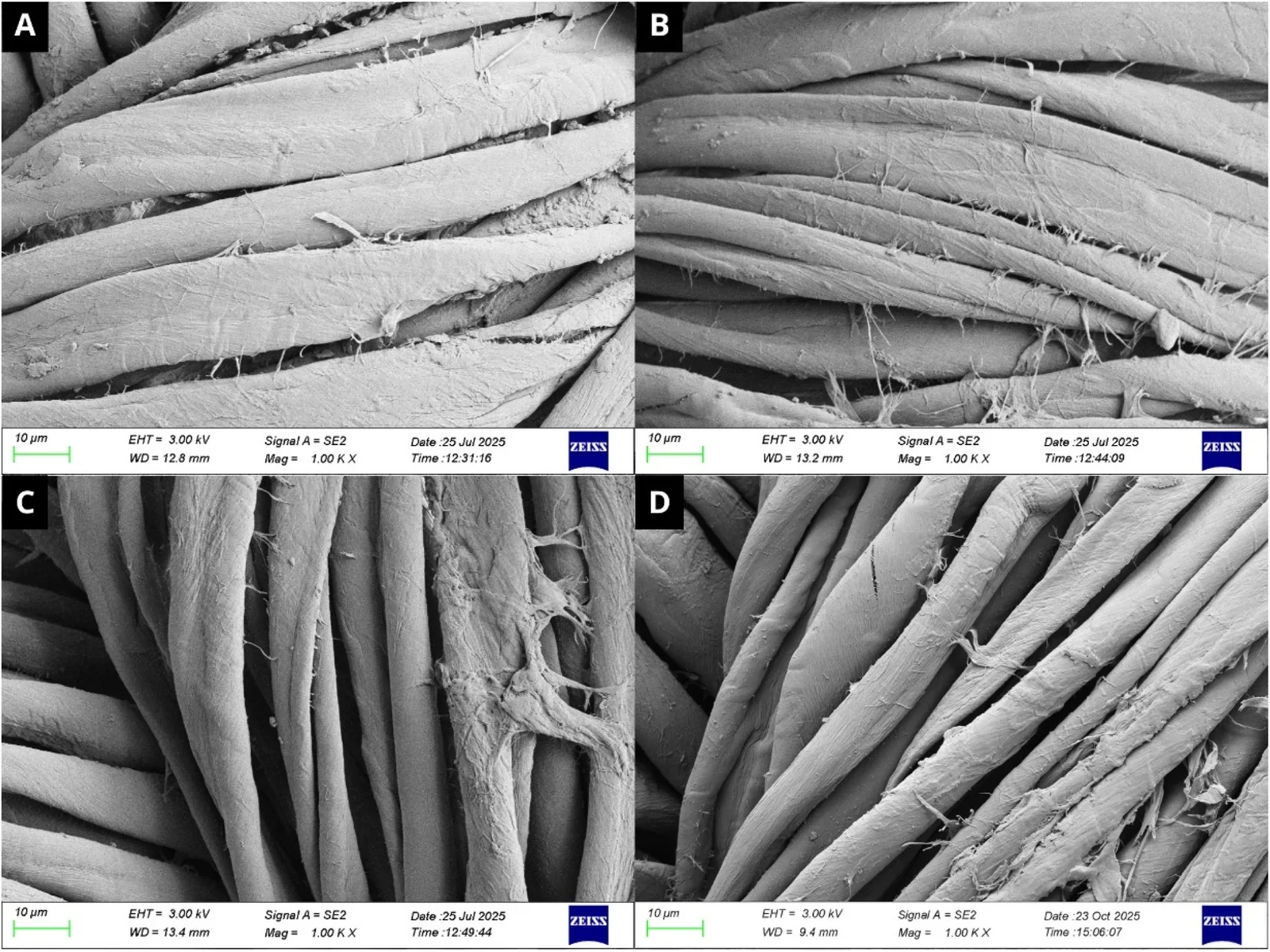
SEM micrographs of (A) fabric before decolorization, after decolorization with (B) Na_2_CO_3_, (C) in a two-bath Na_2_CO_3_/Na_2_S_2_O_4_ process, and (D) in a one-bath Na_2_CO_3_/Na_2_S_2_O_4_ process.

### 3.5 FTIR analysis

The achieved lightness and decolorization results under optimized conditions indicate that the covalently bonded reactive dye molecules could be effectively removed and/or destroyed. This was further examined using Fourier transform infrared spectroscopy (FTIR), which offers insights into the structural modifications in the cotton substrates caused by decolorization. The FTIR spectra of the samples and the FTIR difference spectra are presented in Fig. 8.

**Fig. 8:**
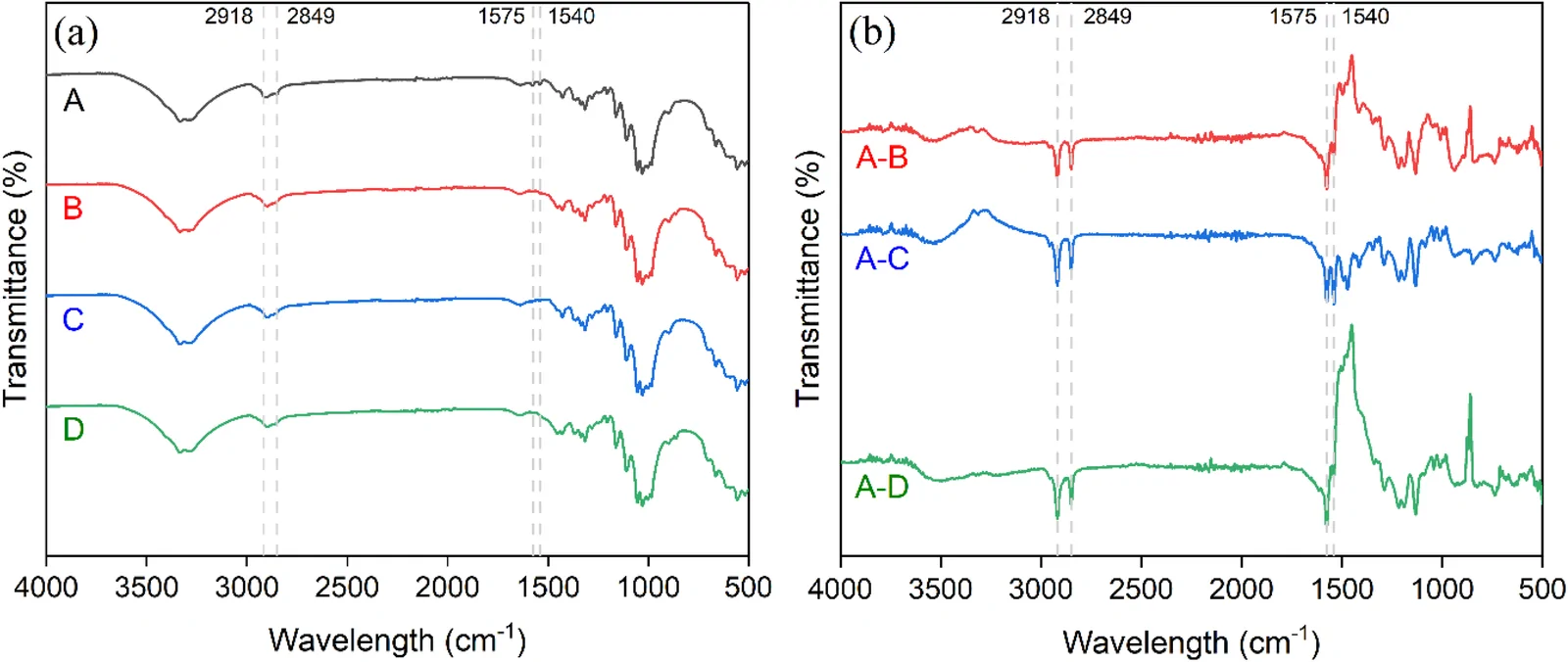
(a) FTIR spectra of the reactive-dyed black fabric (A) before decolorization, and after (B) alkaline hydrolysis (Na_2_CO_3_), (C) sequential two-bath, and (D) sequential one-bath decolorization processes (Na_2_CO_3_/Na_2_S_2_O_4_). (b) FTIR difference spectra highlighting the changes occurring due to decolorization, obtained by subtracting the spectra of the decolorized samples (B–D) from that of the untreated black cotton fabric (A).

The broad band around 3300 cm^−1^ can be attributed to hydrogen-bonded O–H stretching resulting from both inter- and intramolecular bonding of the cotton macromolecules, along with N–H stretching of the fixed dye molecules.^32,33^ This band shows only minor modifications after decolorization, indicating excellent retention of the fabric's mechanical properties.^34^ A broad absorption band from 2800 to 3000 cm^−1^ corresponds to C–H stretching of aliphatic hydrocarbons. The peaks at 2918 and 2849 are characteristic of asymmetric and symmetric stretching vibrations of methylene (–CH_2_^−^) groups present in long alkyl chains.^35^ The intensity of these methylene peaks indicates the presence of reactive dyes on the fabric. The appearance of distinct peaks at 2918 and 2849 cm^−1^ in the FTIR difference spectra implies successful removal of dyes. The most pronounced changes between the samples are observed from 1640 to 1350 cm^−1^. The N=N double bond stretching vibrations appear between 1600 and 1450 cm^−1^, with noticeable changes after decolorization.^36^ The increased intensity of these bands in the decolorized fabrics suggests angular deformation of azo groups, contributions from the –NH_2_ groups of primary amines, and C–N bond vibration in amine groups. Distinctive peaks at 1575 and 1540 cm^−1^ in the untreated black fabric spectrum are associated with N=N stretching of the azo bond and the presence of amine groups, respectively.^37^ The absence of these peaks in the spectra of decolorized fabrics again indicates that the dye molecules have been removed and destroyed. This can also be seen in the FTIR difference spectra, where prominent peaks are present at these wavelengths. A significant increase in intensity at 1450 cm^−1^ is observed after decolorization with sodium carbonate and the one-bath process. This band is generally assigned to the stretching vibrations of –C=C– in substituted benzene and naphthalene rings of reactive dye molecules.^32^ As sodium carbonate primarily facilitates dye removal rather than degradation, the increased peak heights may be due to unbonded dye molecules on the fiber surface and the formation of alkyl groups on the fibers. In addition, the strong peak at 1450 along with a peak at 860 cm^−1^ may be attributed to the presence of Na_2_CO_3_, likely resulting from incomplete washing.^38^ These peaks are absent in the A–C difference spectrum (after the two-bath process), where two washing steps and a separate reductive treatment are applied. In contrast, these peaks are clearly present in the A, B and A–D spectra, indicating residual Na_2_CO_3_ on the fabric surface after alkaline hydrolysis and the one-bath decolorization process. The band at 1132 cm^−1^ is characteristic of the stretching vibration of the C–O–R covalent bond between cotton fiber and dye molecules.^39^ The presence of this band in the FTIR difference spectra indicates cleavage of these covalent dye-fiber bonds. This peak may also be attributed to S=O stretching of SO_3_Na groups presents in vinyl sulfone dyes.^32,37^ Overall, the FTIR results provide valuable insight into the structural modifications in the fabrics following decolorization, particularly highlighting the presence of amino and methylene functional groups.

### 3.6 Depolymerization behavior

Table 4 indicates a substantial reduction in the degree of polymerization (DP) from 3395 to 1861–1883 across all treated samples, corresponding to approximately a 40–45% decrease, while the limiting viscosity decreases proportionally from 1099 to 696–702 mL g^−1^. The marginal differences between alkaline hydrolysis, two-bath, and one-bath treatments suggest that Na_2_CO_3_-induced cellulose chain cleavage is the dominant depolymerization mechanism, while the addition of Na_2_S_2_O_4_ contributes only negligible additional degradation. Alkaline hydrolysis primarily leads to cellulose depolymerization through cleavage of glycosidic bonds.^40^ Besides, treatment parameters can also have a substantial effect on the structural properties of the treated fabrics; prolonged exposure at elevated temperature and pressure can contribute to cellulose chain scission.^7,41^ In contrast, sodium dithionite mainly acts as a selective reducing agent for dye chromophores and has a negligible effect on the cellulose backbone under the studied conditions.^16^ Consequently, in the two-bath process, the DP remains comparable to that obtained after alkaline hydrolysis, indicating that polymer chain scission is dominated by the alkaline step. In the one-bath system, although Na_2_S_2_O_4_ still primarily targets chromophores, its simultaneous presence with Na_2_CO_3_ may enhance fiber swelling and accessibility, leading to slightly increased depolymerization compared to the two-bath process,^10^ though the difference remains marginal.

**Table 4:** Viscosity and degree of polymerization values of the decolorized black and undyed white fabrics

Fabric type	Limiting viscosity (mL g^−1^)	DP
Undyed cotton	1099 ± 4	3395 ± 16
Alkaline hydrolyzed cotton (Two-bath; 1st bath)	702 ± 2	1883 ± 8
Na_2_S_2_O_4_ treated cotton (Two-bath; 2nd bath)	700 ± 3	1875 ± 11
Na_2_CO_3_–Na_2_S_2_O_4_ treated cotton (One-bath)	696 ± 1	1861 ± 4

Despite this significant DP reduction, the relatively low weight loss (≤8.1%) and high tensile strength retention (>94%), as shown in Fig. 6, can be explained by considering the hierarchical structure of cellulose fibers, where chain scission occurs predominantly in amorphous regions. Such molecular-level degradation significantly reduces DP without proportional mass loss, as degraded fragments are largely retained within the fiber matrix, while actual weight loss mainly reflects the limited dissolution of low-molecular-weight fractions. Similarly, tensile strength is governed more by the integrity of the crystalline regions; thus, moderate depolymerization in amorphous regions does not proportionally lead to mechanical deterioration.^42,43^ This decoupling between molecular degradation and macroscopic properties may explain why significant reductions in DP and viscosity can coexist with minimal weight and strength losses.

### 3.7 Elemental analysis

Elemental analysis was used to evaluate decolorization efficiency by quantifying nitrogen and sulfur, which are absent in untreated cotton and therefore indicate the presence of reactive dyes. As shown in Table 5, cotton samples predominantly contained carbon and hydrogen, while the dyed black fabric contained 0.41% nitrogen and 0.46% sulfur due to bonded dye molecules. After decolorization, no sulfur was detected in any sample, suggesting effective dye removal during alkaline hydrolysis; however, slight residual coloration (Table 3) implies that trace dye may remain below the sulfur detection limit (0.02%) of the thermal conductivity detector. The nitrogen content decreased from 0.41% to 0.01% after the first alkaline treatment with Na_2_CO_3_. Nitrogen remained at 0.01% after the reductive step, and no sulfur was detected in any treated sample. These results indicate that alkaline hydrolysis removes most dye molecules by cleaving dye–fiber bonds and transferring them to the decolorization liquor, whereas as the subsequent reductive treatment primarily decolorizes residual dye by disrupting chromophore structures rather than eliminating remaining bonded dye molecules. Although residual nitrogen was detected in decolorized samples, it may not necessarily be due to remaining dyes, as even bleached white cotton fabric can contain small amounts of nitrogen.^44^

**Table 5:** Changes in the elemental composition of dyed and color-stripped samples^a^

Samples	Elements (%)			
	C	H	N	S
Dyed black	43.87	6.17	0.41	0.46
Alkaline hydrolyzed cotton (Two-bath; 1st bath)	41.52	6.05	0.01	ND
Na_2_S_2_O_4_ treated cotton (Two-bath; 2nd bath)	41.58	5.98	0.01	ND
Na_2_CO_3_–Na_2_S_2_O_4_ treated cotton (One-bath)	41.54	6.03	0.01	ND

a [ND: ot detected (below detection limit 0.02%)].

### 3.8 Thermogravimetric analysis

All samples (Fig. 9) exhibited a minor weight loss below 100 °C, attributed to the evaporation of absorbed moisture and trapped gases.^45^ The principal thermal degradation of cellulose in decolorized fabrics occurred between 330–380 °C, characterized by a sharp and narrow weight-loss slope. In contrast, the black-dyed fabric showed an earlier degradation onset at 287 °C and a broader slope, indicating altered thermal behavior. Cellulose decomposition involves depolymerization, dehydration, and glycosyl unit breakdown, leading to char formation.^46^ The decolorized fabrics produced only 9.2 to 9.7% char residue at 400 °C, whereas the dyed fabric yielded 24.0%, demonstrating that reactive dyes reduce the decomposition temperature and promote solid residue formation.^47^ A further minor weight loss above 500 °C corresponds to oxidation of the char into low-molecular-weight gaseous products due to the presence of small quantities of oxygen.^46^ The altered pyrolysis behavior of dyed cotton is linked to covalent bonding between reactive dyes and cellulose anhydroglucose units. Following dye removal, the onset degradation temperatures increased, and the thermal degradation behavior of the decolorized fabrics closely resembled that of undyed cotton.^42,48^

**Fig. 9:**
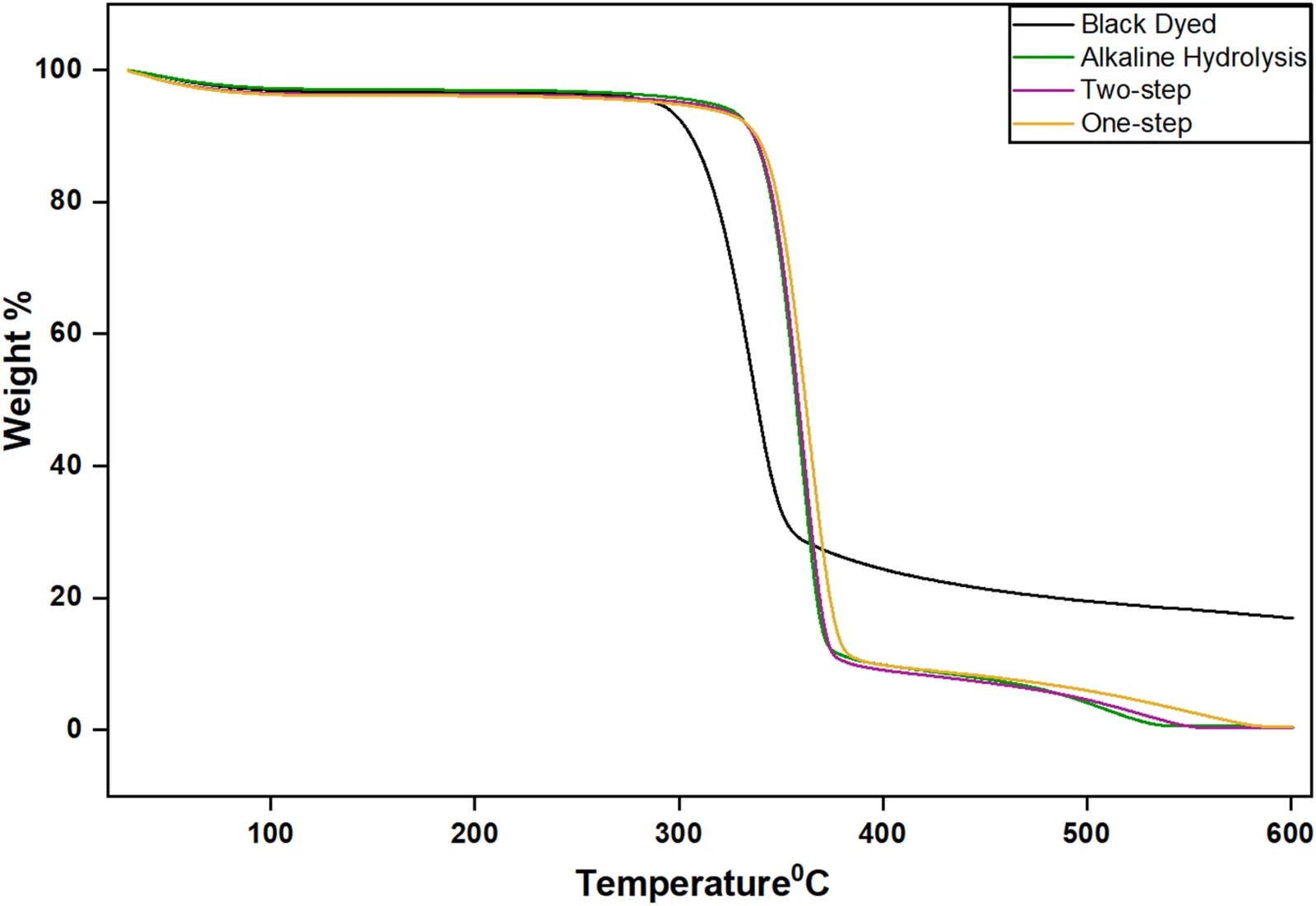
TGA curves of the original and treated black fabric.

### 3.9 Crystallinity analysis

The X-ray diffraction patterns of the cotton fabric are presented in Fig. 10. The structure exhibits characteristic diffraction peaks at 2*θ* values of 15°, 16°, 22° and 34°, corresponding to (1–10), (110), (200), and (004) planes, respectively.^49^ The shape of the spectra has not changed with the decolorization treatments, implying that the crystal structure remained preserved cellulose I crystalline structure during the treatments. However, the peak at 2*θ* = 22° slightly increases in intensity with the removal of the dye molecules. This peak represents the sum of the crystalline and amorphous components, while amorphous-only components are represented by the intensity at the minimum between peaks (110) and (200), at around 2*θ* = 18°.^50^ In general, the sharp and strong peaks can be attributed to the crystalline regions and the broader signals to the amorphous regions in cellulose.^51^

**Fig. 10:**
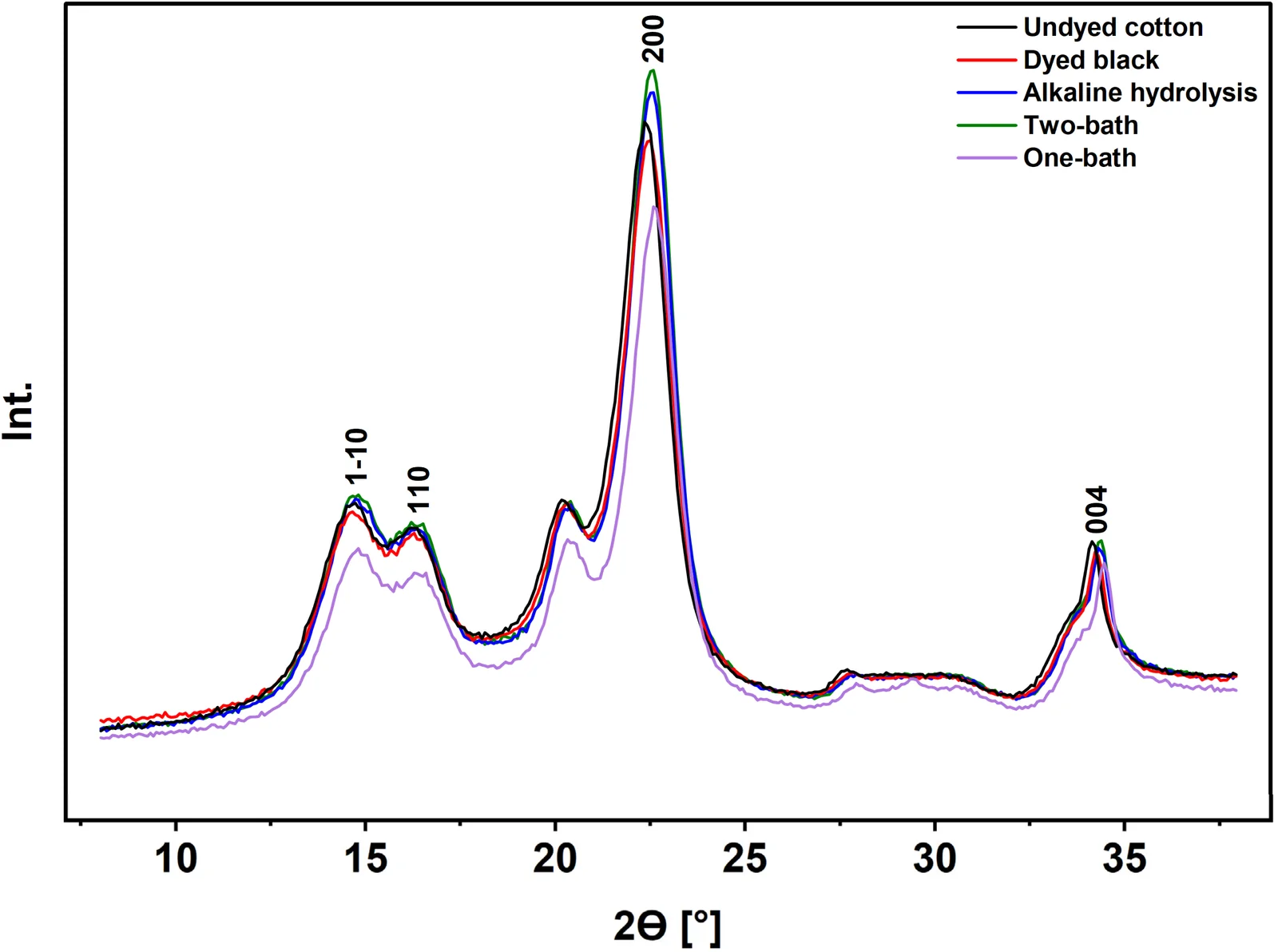
X-ray diffraction patterns of the undyed cotton fabric as well as the dyed black cotton fabric before and after alkaline hydrolysis, two-bath, and one-bath decolorization treatments.

The variation of peak intensity and the area under the peaks is associated with distinct structural changes in the cellulose molecule. As the peak (200) at 2*θ* = 22° has clearly increased in intensity after decolorization, but the amorphous-only region at 2*θ* = 18° has remained roughly at the same intensity, it can be deduced that the number of crystalline regions has increased. This may be due to alkali attacking the amorphous regions in cellulose more readily than the crystalline regions. Due to the degradation of amorphous regions, possibly through both peeling reactions and scission, the proportion of crystalline regions increases. However, the reaction is relatively slow, which is why a remarkable increase in crystallinity is not observed.^52^ In addition, the decolorization treatments slightly caused shifting of the peak (200) at 2*θ* = 22° to a higher 2*θ* degree due to the partial removal of non-cellulosic material, in this case, the reactive dyes.^53^ According to Bragg's law (*nλ* = 2*d* sin *θ*), the shift of (200) results in a decreased *d*-spacing, indicating a denser crystal structure, especially in the (200) plane.^54^

Table 6 shows the crystallinity index values measured using two different methods: the Savitzky–Golay, which considers the peak area, and the Segal method, which estimates the crystallinity by comparison of intensities. According to both methods, the crystallinity index increases after decolorization, supporting the interpretation of Fig. 10. Due to the overall increase in the peak area after treatment, the crystallinity index increased roughly 5 percentage points when measured using the Savitzky–Golay filter, while the Segal method reports an increase of two percentage points. The alkaline hydrolysis treatment had the most significant effect on the crystallinity of the fabric, as seen in the measured crystallinity index values (Table 6). Thus, the slight increase in crystallinity may have resulted from preferential degradation of amorphous regions during alkaline treatment. These regions are more accessible and reactive, so their partial removal increases the relative proportion of crystalline domains, leading to a higher calculated crystallinity index. At the same time, dye removal can also contribute to this effect. Reactive dyes are primarily located in the amorphous regions of cellulose, and their removal can reduce structural disorder and enhance the apparent ordering of the fiber.

**Table 6:** Savitzky–Golay (SG) and Segal crystallinities of the undyed, dyed and decolorized fabrics

Sample	CI [%] SG	CI [%] Segal
Undyed cotton	45 ± 2	80 ± 1
Dyed cotton (black color)	45 ± 2	80 ± 1
Alkaline hydrolyzed cotton (Two-bath; 1st bath)	50 ± 2	82 ± 1
Na_2_S_2_O_4_ treated cotton (Two-bath; 2nd bath)	50 ± 2	82 ± 1
Na_2_CO_3_–Na_2_S_2_O_4_ treated cotton (One-bath)	50 ± 2	82 ± 1

### 3.10 One-bath scale-up

Based on the results achieved at the laboratory scale, a scale-up in an air bath digester (Haato Oy, Finland) was performed to assess the decolorization performance on a large scale. The sequential one-bath process was chosen for scale-up experiments due to its efficient color removal, accompanied by minimal fiber damage. The colorimetric properties and percentage weight loss results are presented in Fig. 11, and the numerical data can be found in Table S4. The scale-up results demonstrate that the one-bath Na_2_CO_3_/Na_2_S_2_O_4_ process has the potential to become an industrially feasible decolorization process for reactive-dyed cotton. All scaled-up samples either reached or exceeded the lightness, decolorization, and whiteness index values obtained at the laboratory scale. As seen in Fig. 11(a–c), the colorimetric properties of the green and grey fabrics were clearly enhanced when the process was scaled up. Additionally, the percentage weight loss significantly decreased for grey, navy, and green fabrics (Fig. 11d). The least difference in weight loss results was observed in black fabric, which is likely due to its high dye concentration that contributes to the weight loss results. The one-bath decolorization system was thus demonstrated to be an effective method for reactive-dye removal from cotton fabrics and has potential for large-scale production. The resulting fabrics were uniformly decolorized despite the absence of sufficient agitation.

**Fig. 11:**
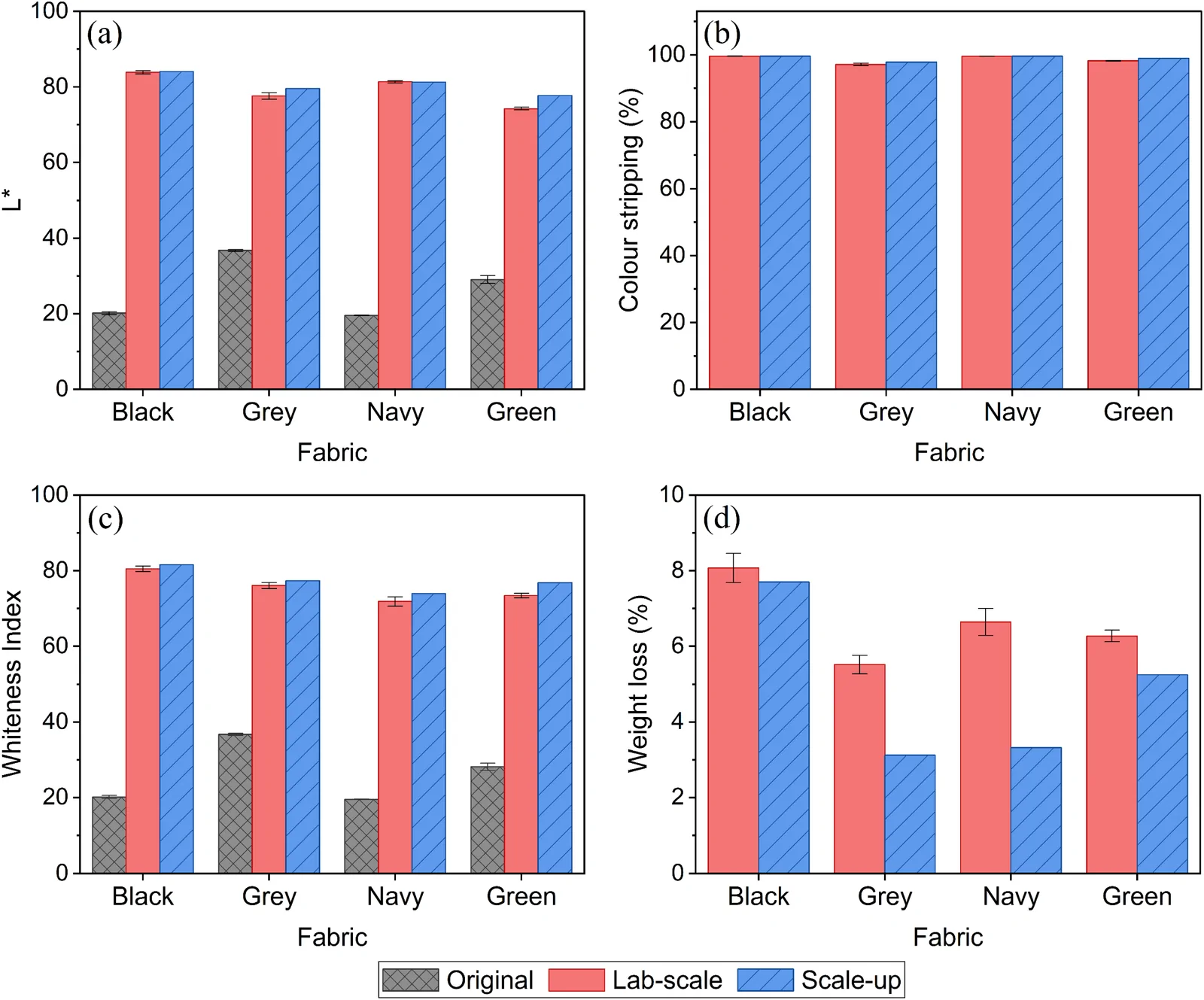
The (a) lightness (L*), (b) color stripping, (c) whiteness index, and (d) weight loss results of the lab-scale and scaled-up one-bath (Na_2_CO_3_/Na_2_S_2_O_4_) decolorized fabrics.

### 3.11 Decolorization mechanism of reactive-dyed cotton fabric

The decolorization of reactive-dyed cotton under alkaline and reductive conditions can be explained by a combination of chemical and physical processes. During the alkaline hydrolysis step, the elevated pH and temperature promote fiber swelling, which increases the accessibility of the fiber structure.^17^ Under these conditions, the covalent bonds between the reactive dye molecules and the hydroxyl groups of cellulose are cleaved, converting the bound dye into its hydrolyzed (non-reactive) form. This facilitates the release of dye molecules from the fiber matrix. In addition, alkaline conditions may induce partial degradation or modification of the dye chromophore, particularly in dyes containing reactive groups or labile substituents, contributing to decolorization. In the subsequent reductive stage, sodium dithionite acts as a reducing agent that may disrupt the chromophore systems of the residual dyes. In particular, reduction of azo (–N=N–) linkages or other conjugated structures leads to a loss of color by breaking the extended π-electron system responsible for light absorption.^10^ This step primarily contributes to decolorization rather than complete dye removal. The proposed reaction scheme illustrating the proposed mechanism by which sodium carbonate and sodium dithionite facilitate the cleavage of the covalent bond between the reactive dye molecules and cellulose fibers during the decolorization process is demonstrated in Fig. 12. To further support this mechanism, a UV-visible spectroscopic technique was performed to measure absorbance against wavelength for different decolorized filtrates. From the test results, Na_2_CO_3_ filtrates in two-step processes exhibited distinct absorbance peaks, which can be attributed to the presence of dissolved dye molecules that remained in the decolorized filtrates after Na_2_CO_3_ treatment. On the other hand, Na_2_S_2_O_4_ filtrates were less colored and showed no characteristic dye absorbance. The less colored filtrates of Na_2_S_2_O_4_ also suggest that Na_2_S_2_O_4_ can induce the reduction of the dye chromophore, resulting in less colored filtrates and fabrics close to white. The filtrates of the one-bath decolorization method showed distinct peaks at certain wavelengths. This is mainly due to cleavage of the covalent bond between dyes and cellulose after Na_2_CO_3_ treatment, releasing a fraction of dye molecules into the decolorization liquor. Thus, the UV-vis results are consistent with the observed color changes both in decolorized filtrates and treated fabrics, thereby supporting the proposed mechanism. Absorbance peaks of one-bath and two-bath decolorized filtrates against wavelength and visual appearance of different decolorized filtrates are illustrated in Fig. S4.

**Fig. 12:**
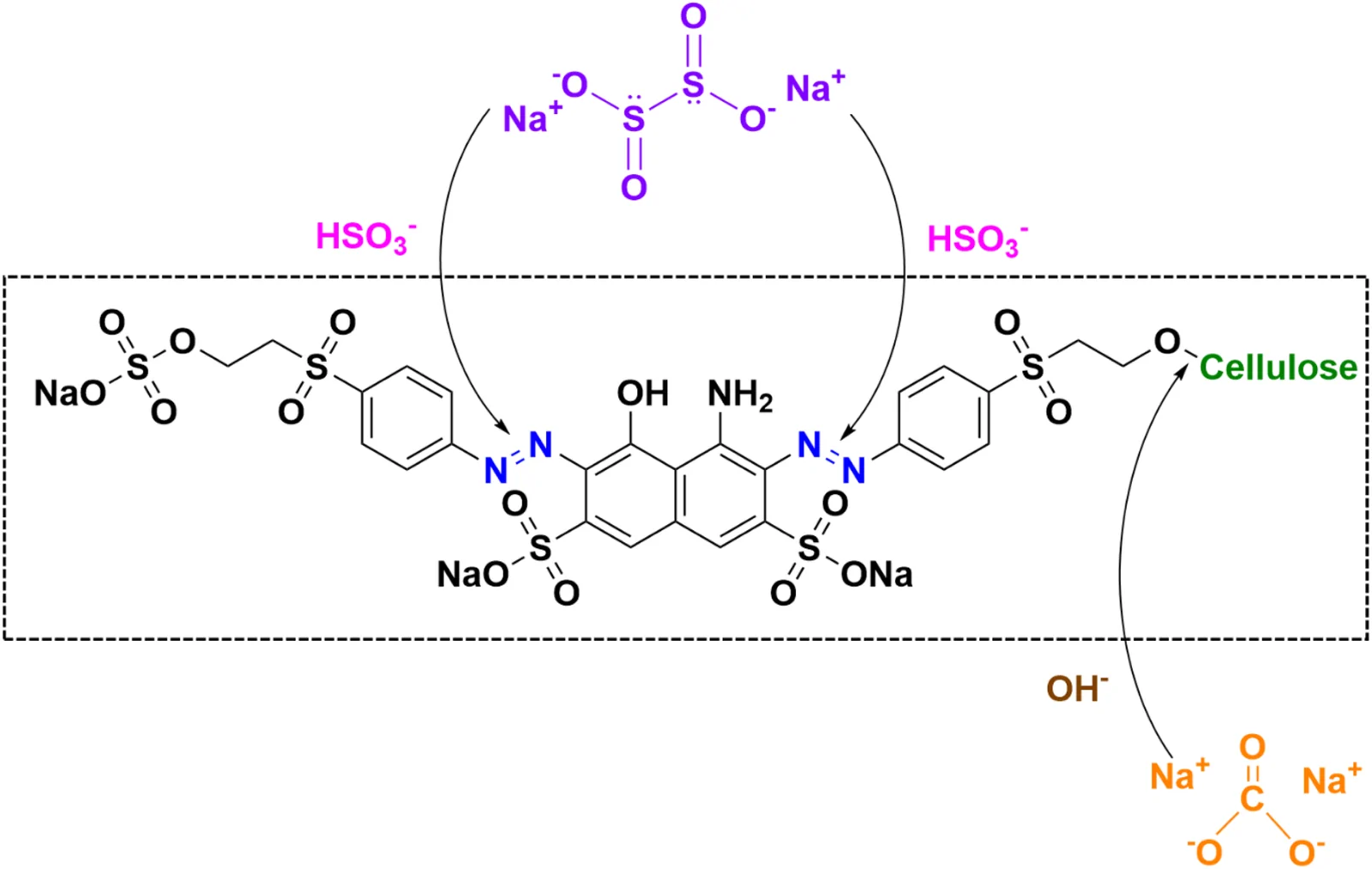
Proposed decolorization pathways involving sodium carbonate and sodium dithionite. The arrows indicate potential sites of chemical attack.

### 3.12 Comparison between the one-bath and the two-bath processes

According to the colorimetric results, the one-bath process provided higher lightness, decolorization, and whiteness index values compared to the two-bath process. The color of the two-bath decolorized fabrics remained like the original, whereas the one-bath decolorized fabrics had clearly changed color. The biggest difference in lightness was detected with the grey fabric, as the lightness difference between the one-bath and two-bath processes was roughly 14. For other fabrics, the difference was a maximum of 6 units but was visually more significant due to the changes in color of the fabrics. Uneven decolorization was observed during the optimization of the two-bath process, as well as for some colored fabrics decolorized with the optimized process. The one-bath process did not produce any unevenly decolorized fabrics; hence, the pH of the decolorization solution evidently has a considerable effect on the decolorization efficiency and performance of sodium dithionite since the other treatment conditions (concentration, temperature, time, and mass-to-liquor ratio) in both methods were identical. In the two-bath process, the starting pH of the Na_2_S_2_O_4_ liquor was roughly pH 6 and dropped to around pH 3–4 during the process, depending on the dyed fabric. For the one-bath process, the start and end pH values for Na_2_S_2_O_4_ decolorization were approximately pH 10.4–10.8 and pH 10.1, respectively. Thus, it can be deduced that sodium dithionite performs better in an alkaline environment. This is in line with previous studies that have demonstrated effective reactive dye decolorization with sodium dithionite at pH values ranging from 9 to 13.7.^10,33^ In the presence of Na_2_CO_3_, the cotton fibers swell and enable Na_2_S_2_O_4_ to diffuse more effectively in the fiber matrix,^8^ which can explain the enhanced decolorization performance in the one-bath process. In addition to better decolorization performance, the one-bath process saved time and resources. The amount of decolorization liquor needed was halved as no second decolorization bath was involved. Using a single bath also allows sodium dithionite to decolorize the filtrate to some extent, which can reduce wastewater treatment costs. Additionally, significant freshwater and cost savings derive from the absence of the washing step between the alkaline hydrolysis and reductive decolorization treatments. As up to half of the total costs of reactive dyeing come from the washing and wastewater treatments,^55^ it can be assumed that they are a significant expense in reactive dye decolorization as well. Therefore, the proposed one-bath method can bring considerable savings not only in process costs, but also in freshwater and energy consumption. As the physicophysical–mechanical properties after the one-bath and two-bath processes reveal, their effects on the mechanical properties of the fabrics do not differ considerably. Both methods provided excellent tensile strength retention and caused no increased degradation in viscosity and DP when applied after the alkaline hydrolysis treatment. Additionally, neither of the methods caused crystal structure changes nor considerable fiber surface degradation while efficiently removing dyes from the fabrics. While both methods caused practically no additional degradation on the cotton fabrics after alkaline hydrolysis, the colorimetric properties had major differences. The overall evaluation of two decolorization processes is stated in Table 7.

**Table 7:** Overall evaluation of two decolorization processes^a^

Evaluation parameters	Two-bath	One-bath
Chemical consumption	*	*
Energy consumption	***	*
Water consumption	***	*
Decolorization performance	**	***
Chance for scalability	**	***
Environmental impact	***	*
Flexibility in process control	***	*

a * = Low, ** = medium, *** = high.

To produce 1 kg of white feedstock, the two-bath decolorization process requires 1.105 kg of waste fibers, 8.840 kWh of electricity, 243.094 kg of water, 0.221 kg of Na_2_CO_3_, 0.022 kg of Na_2_S_2_O_4_, and 0.088 kg of soap. In comparison, the one-bath decolorization process requires 1.122 kg of waste fibers, 6.734 kWh of electricity, 134.680 kg of water, 0.224 kg of Na_2_CO_3_, 0.022 kg of Na_2_S_2_O_4_, and 0.045 kg of soap. Thus, the one-bath process uses substantially less electricity, water, and soap, while the amounts of Na_2_CO_3_ and Na_2_S_2_O_4_ remain identical between the two processes.

The wastewater generated from the two-bath (alkali hydrolysis followed by reduction) and one-bath decolorization processes using Na_2_CO_3_ and Na_2_S_2_O_4_ was evaluated in terms of chemical oxygen demand (COD), biological oxygen demand (BOD), total organic carbon (TOC), and adsorbable organic halogen (AOX). For the two-bath process, the alkaline hydrolysis step using Na_2_CO_3_ produced wastewater with a COD of 2987 ± 12 mg L^−1^, BOD of 594 ± 41 mg L^−1^, TOC of 1043 ± 21 mg L^−1^, and AOX of 3.35 mg L^−1^. The subsequent reduction step using Na_2_S_2_O_4_ resulted in COD, BOD, TOC, and AOX values of 993 ± 12, 515 ± 9, 35 ± 1, and 4.19 mg L^−1^, respectively. In comparison, the one-bath treatment using Na_2_CO_3_ and Na_2_S_2_O_4_ generated wastewater with a COD of 4420 ± 269 mg L^−1^, BOD of 1080 ± 47 mg L^−1^, TOC of 1058 ± 10 mg L^−1^, and AOX of 3.78 mg L^−1^. The wastewater analyses indicate that the one-bath treatment produces COD, BOD, and TOC values comparable to the combined wastewater streams generated during the two-bath treatment, while the AOX concentration remains low. Importantly, the proposed one-bath process eliminates the need for a separate alkali-hydrolysis and reduction bath, thereby simplifying the treatment sequence and potentially reducing water consumption, process time, and the number of wastewater streams requiring handling. It could be interpreted in the context of the overall process, the two-bath process generates two separate wastewater streams.

Sodium carbonate and sodium dithionite can potentially be recovered from the wastewater generated and reused, thereby reducing chemical consumption and improving process sustainability. Existing industrial wood-pulping processes employ chemical recovery systems that can regenerate alkaline solutions containing sodium carbonate (Na_2_CO_3_) and sodium sulfite (Na_2_SO_3_) at different ratios and pH values.^56^ This suggests that the spent liquor from the proposed one-bath decolorization process could potentially be integrated into existing chemical-recovery schemes, particularly in facilities where suitable recovery infrastructure is already available. In addition, aqueous Na_2_SO_3_ solutions can be converted electrochemically to sodium dithionite (Na_2_S_2_O_4_), providing a potential route for regenerating the reducing agent.^57^ Thus, in principle, the process could be designed to recover and reuse the inorganic reagents, thereby reducing fresh chemical consumption and improving the overall resource efficiency of the treatment.

## 4 Conclusions

The study successfully demonstrates that reactive dyed post-consumer cotton waste can be decolorized through Na_2_CO_3_/Na_2_S_2_O_4_ treatments. The decolorized cotton achieved a high quality that makes it suitable for mechanical recycling. If the intrinsic viscosity is further reduced through acid treatment, it can also be used for chemical recycling. The continuous one-bath process performed better in terms of fabric lightness and uniformity, as well as water and energy consumption. Key findings of the study are highlighted as:

• ≥97% decolorization and L* >80 achieved, one-bath yielded higher L* and uniformity *vs.* two-bath.

• The decolorized cotton shows excellent strength retention and low weight loss.

• Intrinsic viscosity and degree of polymerization mainly drop during the alkaline step, and reductive treatment caused negligible chain breakage.

• XRD confirms that cellulose crystallinity is fully retained.

• SEM images show that the fiber surface remains intact and is unaffected by the process.

• CHNS elemental analysis confirms successful dye removal by pointing to the reduction of nitrogen and sulfur.

• TGA results confirm that the dye removal restored the degradation pattern.

These results reveal that the one-bath process is a scalable and resource-efficient method for producing high-quality cellulose feedstock. It also supports textile circularity by diverting dyed waste from landfills and incineration.

Future research should focus on conducting life-cycle assessment to measure its environmental and economic benefits at the industrial level. The process should also be adapted for blended textiles such as cotton/polyester and for alternative dye types beyond reactive dyes. It is crucial to integrate mechanical spinning and chemical dissolution, such as Lyocell and Ioncell, to confirm full textile-to-textile recycling. Further work should also develop methods to recover, and reuse used chemicals, dyes, and water. AI/ML-based modelling and process optimization could be integrated in the future work.

## Author contributions

Elsa Vuorenmaa: methodology, formal analysis, investigation, data curation, writing – original draft; Md. Reazuddin Repon: conceptualization, methodology, writing – original draft, formal analysis, investigation, data curation, visualization, validation; Tonmoy Saha: software, validation, formal analysis, investigation, writing – original draft, visualization; Shubhajit Dutta: data collection, software, writing – original draft, formal analysis, visualization; Inge Schlapp-Hackl: software, formal analysis, writing – original draft, writing – review & editing; Tapani Vuorinen: conceptualization, methodology, writing – review & editing, supervision, and Ali R. Tehrani-Bagha: conceptualization, methodology, formal analysis, supervision, resources, writing – reviewing and editing, project administration and funding acquisition.

## Conflicts of interest

There are no conflicts to declare.

## Supplementary Material

RA-OLF-D6RA05247A-s001

## Data Availability

The data supporting the findings of this study are available within the article and its supplementary information (SI). Supplementary information: four figures (Fig. S1–S4) and four tables (Tables S1–S4) that provide supporting experimental procedures and characterization data. The figures illustrate the effects of treatment time on colour stripping using alkaline hydrolysis, two-bath, and one-bath processes; the whiteness index of colour-stripped fabrics; the colour strength (*K*/*S*) curves of black, grey, navy, and dark green fabrics following the three stripping treatments; and the absorbance spectra and visual appearance of the stripping filtrates. The tables present the colourimetric data before and after stripping, trace copper detected by X-ray photoelectron spectroscopy (XPS), the tensile strength and retention of treated fabrics, and a comparison of laboratory-scale and scaled-up one-bath stripping processes. See DOI: https://doi.org/10.1039/d6ra05247a.
