# Supplementary material for: Comparative study of decolorization processes for dyed cotton waste: enabling circularity through white feedstock
Source: RSC Adv. 2026 Oct 21. Online ahead of print. doi: 10.1039/d6ra05247a (PMC13684337; doi:10.1039/d6ra05247a)
Supplement: Supplementary file 1 [file RA-OLF-D6RA05247A-s001.pdf]

Supplementary Information (SI) for RSC Advances.
This journal is © The Royal Society of Chemistry

Supporting Information

Comparative study of decolorization processes for dyed cotton waste: Enabling circularity through white feedstock

Elsa Vuorenmaa, Md. Reazuddin Repon, Tonmoy Saha, Shubhajit Dutta, Inge Schlapp-Hackl, Tapani Vuorinen, and Ali R. Tehrani-Bagha*

Department of Bioproducts and Biosystems, School of Chemical Engineering, Aalto University, Vuorimiehentie 1, 02150, Espoo, Finland

*** Corresponding authors:** ali.tehrani@aalto.fi

This supplementary file includes:

Figure S1. Optimization of treatment time for color stripping: effect of time during A) alkaline hydrolysis; B) two-bath process; and C) one-bath process, where $Na_2S_2O_4$ is added directly to the alkaline bath after temperature reduction.

Figure S2. Whiteness Index values of the alkali ([Na_2CO_3] 10 g/L, 130 °C, 60 min, 1:20), two-bath ([$Na_2S_2O_4$] 5 g/L, 60 °C, 60 min, 1:20), and one-bath ([$Na_2S_2O_4$] 5 g/L, 60 °C, 60 min, 1:20) color-stripped fabrics.

Figure S3. Color strength (K/S) curves of A) black (BL), B) grey (GR), C) navy (NV), and D) dark green (DG) fabrics after color stripping treatments: Na_2CO_3 alkaline hydrolysis (-SC), Na_2CO_3□$Na_2S_2O_4$ two-bath treatment (-2), and Na_2CO_3□$Na_2S_2O_4$ one-bath treatment (-1).

Figure S4. a) Absorbance peaks of one bath and two bath stripping filtrates against wavelength b) Visual appearance of different color stripping filtrates.

Table S1. Colorimetric data of the reactive-dyed cotton fabrics before and after alkaline hydrolysis (Na_2CO_3), two-bath ($Na_2CO_3/Na_2S_2O_4$), and one-bath ($Na_2CO_3/Na_2S_2O_4$) color stripping treatments.

Table S2. Traces of copper (Cu) from X-ray photoelectron spectroscopy after decolorisation with Na_2CO_3-$Na_2S_2O_4$

Table S3. The tensile strength (TS) and tensile strength retention (RTS) results of the four reactive-dyed cotton fabrics after alkaline hydrolysis (Na_2CO_3) color stripping (-SC), two-bath Na_2CO_3□$Na_2S_2O_4$ color stripping (-2), and one-bath $Na_2CO_3/Na_2S_2O_4$ color stripping (-1).

Table S4. Comparison of the results of lab-scale and scaled-up one-bath ($Na_2CO_3/Na_2S_2O_4$) color stripping process performed on different color reactive-dyed cotton fabrics.

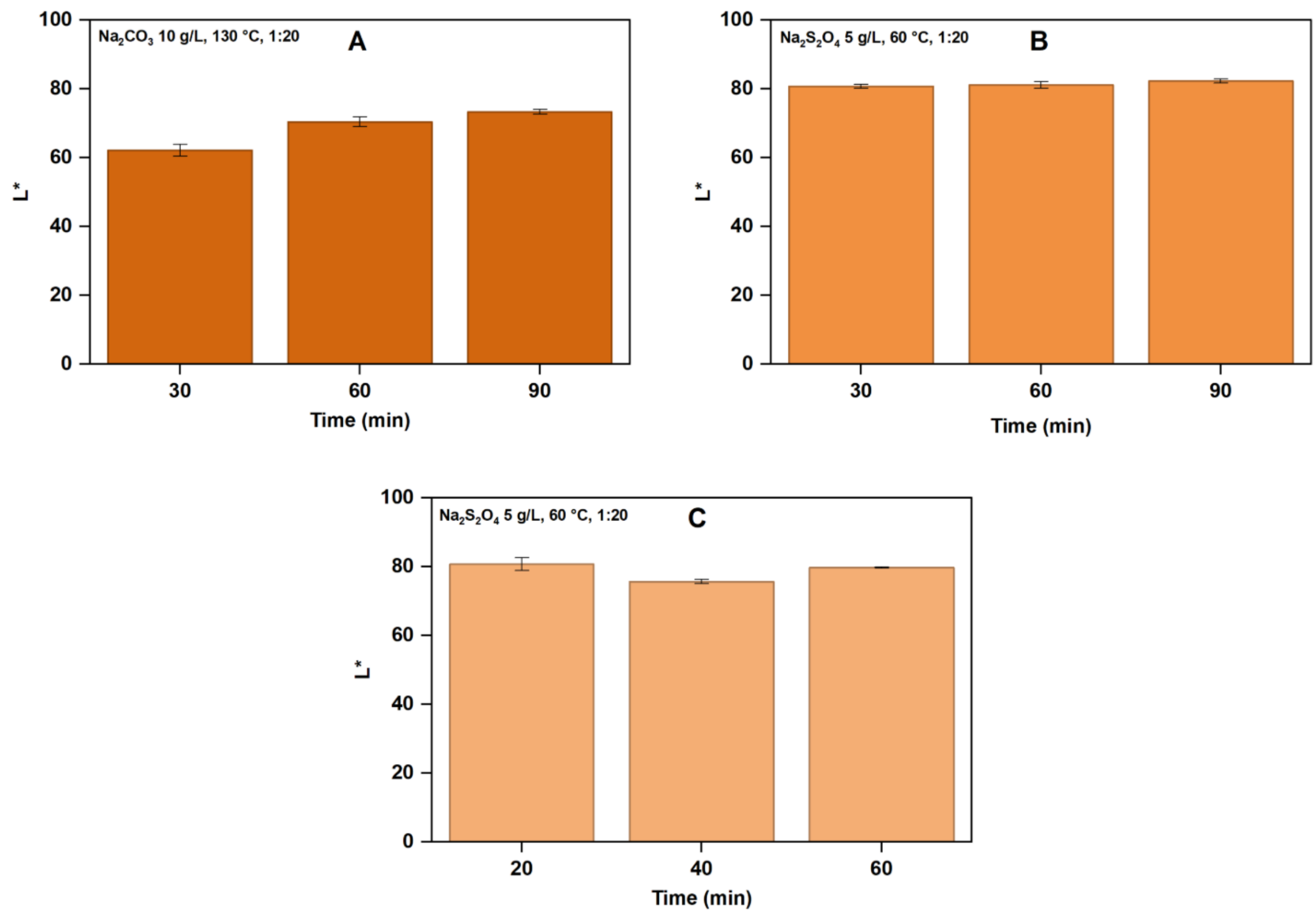

Figure S2. Optimization of treatment time for color stripping: effect of time during A) alkaline hydrolysis; B) two-bath process; and C) one-bath process, where $Na_2S_2O_4$ is added directly to the alkaline bath after temperature reduction.

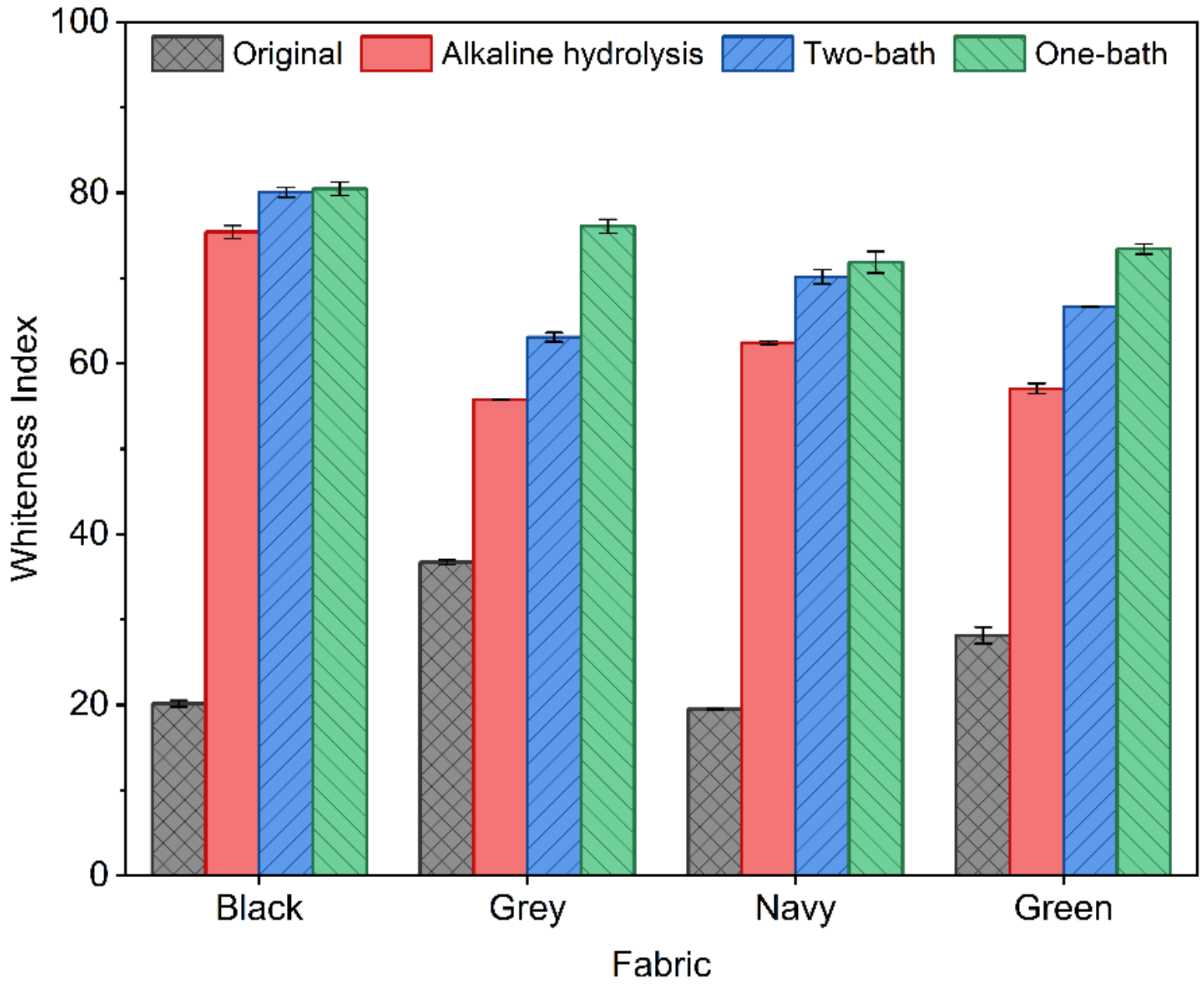

Figure S2. Whiteness Index values of the alkali ([Na_2CO_3] 10 g/L, 130 °C, 60 min, 1:20), two-bath ([$Na_2S_2O_4$] 5 g/L, 60 °C, 60 min, 1:20), and one-bath ([$Na_2S_2O_4$] 5 g/L, 60 °C, 60 min, 1:20) color-stripped fabrics.

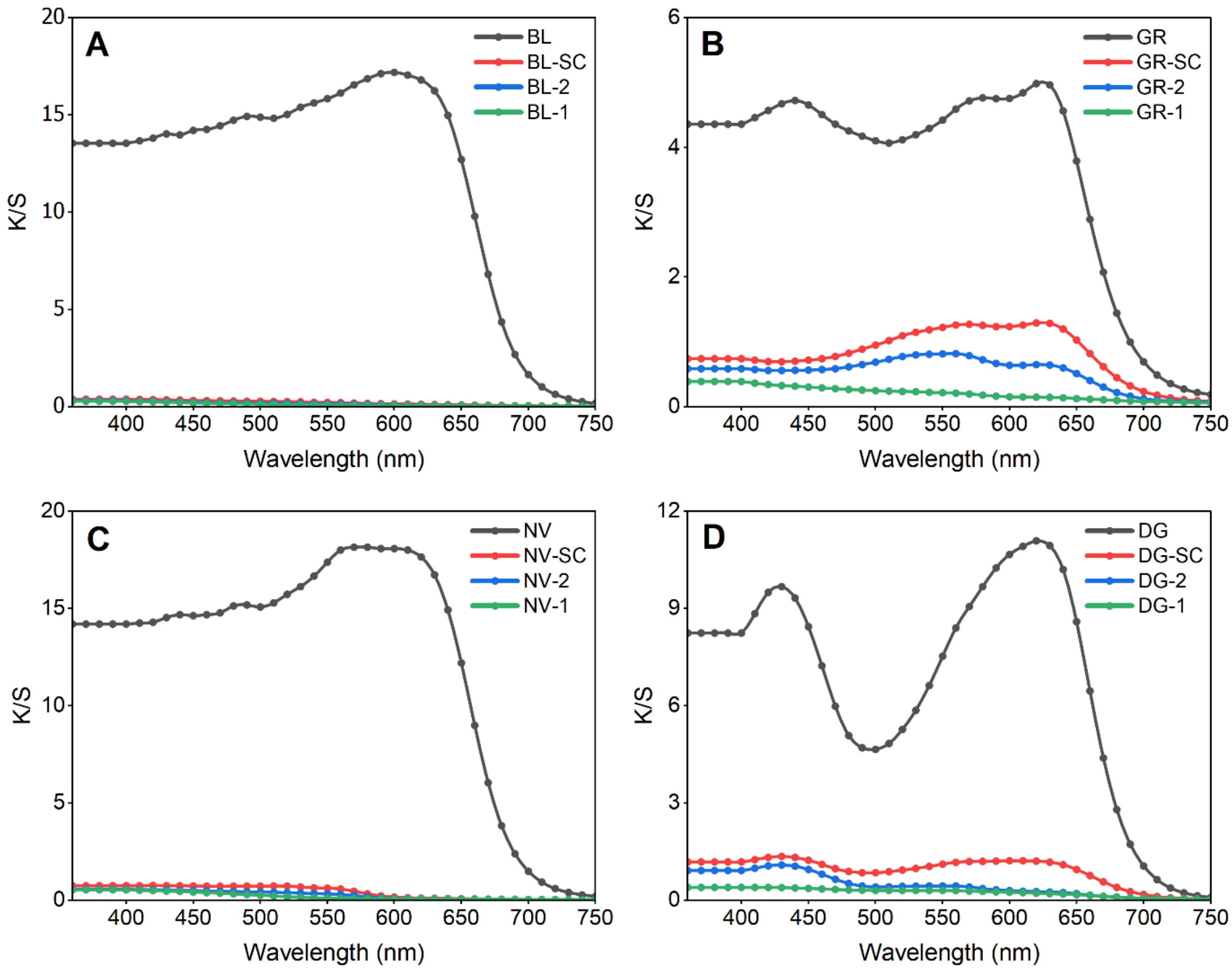

Figure S3. Color strength (K/S) curves of A) black (BL), B) grey (GR), C) navy (NV), and D) dark green (DG) fabrics after color stripping treatments: Na_2CO_3 alkaline hydrolysis (-SC), Na_2CO_3□$Na_2S_2O_4$ two-bath treatment (-2), and Na_2CO_3□$Na_2S_2O_4$ one-bath treatment (-1).

(a)

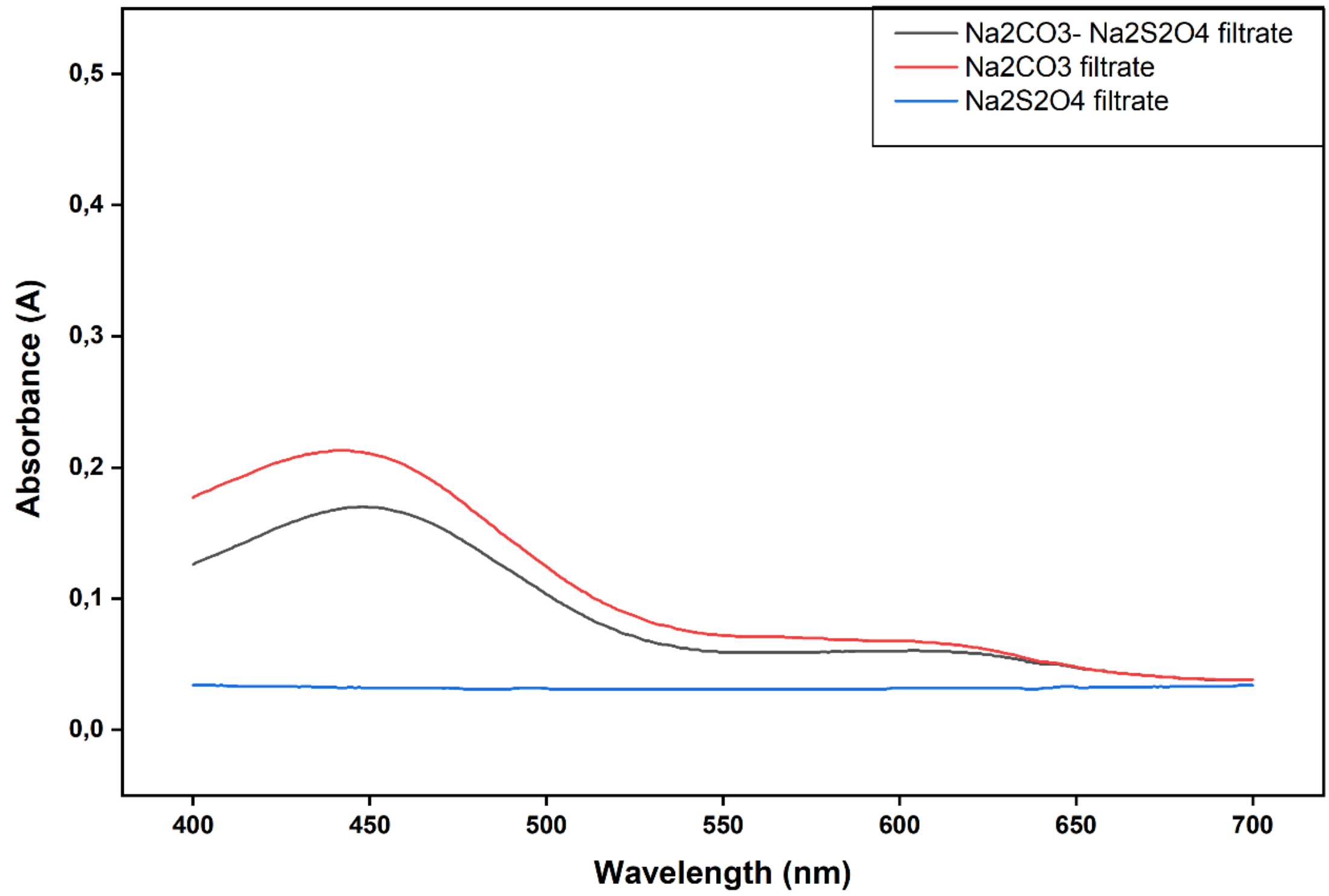

(b)

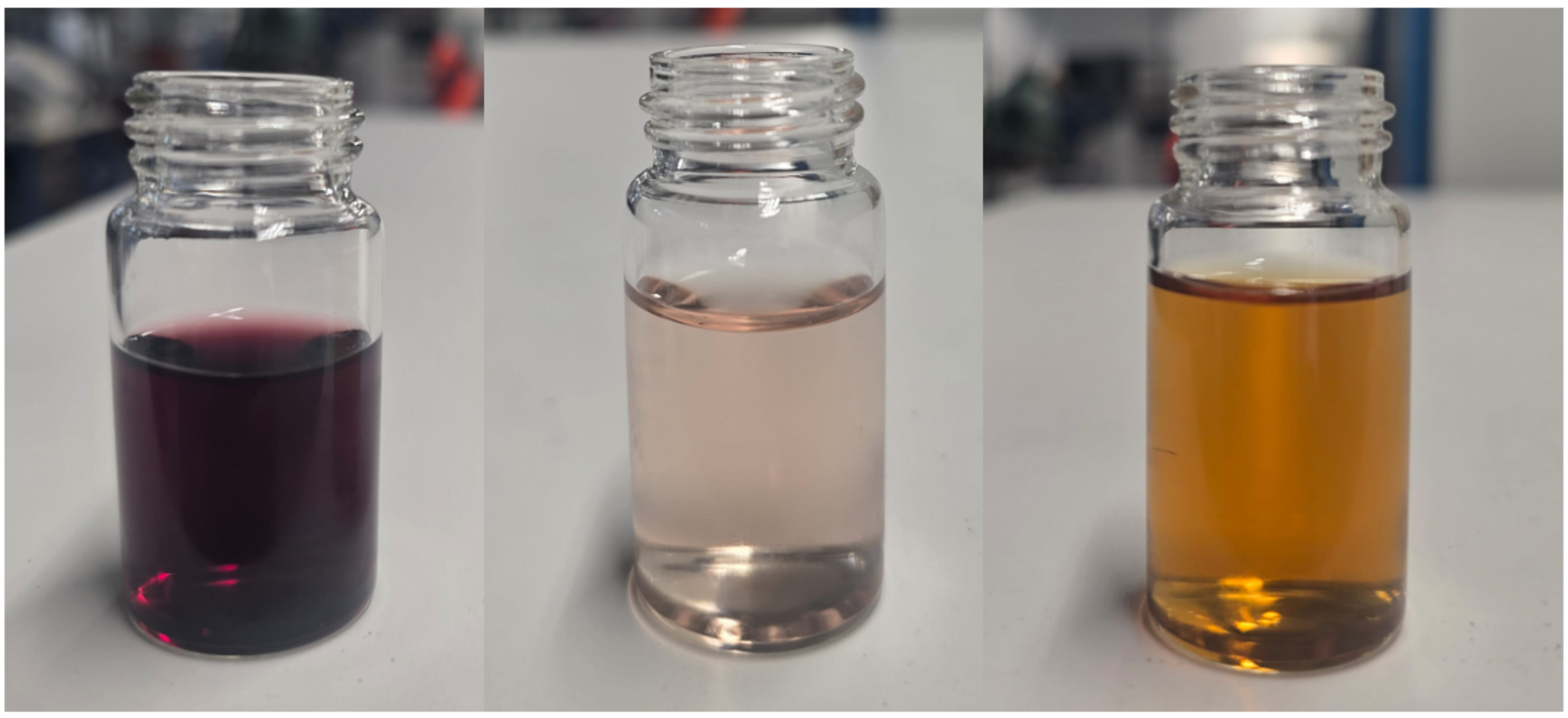

Na_2CO_3 filtrate $Na_2S_2O_4$ filtrate Na_2CO_3- $Na_2S_2O_4$ filtrate

Figure S4. a) Absorbance peaks of one bath and two bath stripping filtrates against wavelength b) Visual appearance of different color stripping filtrates

Table S1. Colorimetric data of the reactive-dyed cotton fabrics before and after alkaline hydrolysis (Na_2CO_3), two-bath ($Na_2CO_3/Na_2S_2O_4$), and one-bath ($Na_2CO_3/Na_2S_2O_4$) color stripping treatments.

Fabric	Treatment	Color Stripping (%)	L*		WI	
			Initial	Treated	Initial	Treated
Undyed cotton	-	-	92.80 ± 0.13	-	91.88 ± 0.13	-
Black	Alkaline Hydrolysis	99.24 ± 0.08	20.18 ± 0.37	77.30 ± 0.95	20.15 ± 0.37	75.42 ± 0.76
	Two-bath	99.58 ± 0.07		82.33 ± 0.74		80.07 ± 0.59
	One-bath	99.59 ± 0.03		83.85 ± 0.41		80.48 ± 0.76
Grey	Alkaline Hydrolysis	84.73 ± 0.61	36.75 ± 0.27	56.93 ± 0.13	36.73 ± 0.28	55.79 ± 0.00
	Two-bath	88.35 ± 0.31		63.76 ± 0.52		63.09 ± 0.55
	One-bath	97.10 ± 0.39		77.64 ± 0.86		76.08 ± 0.80
Navy	Alkaline Hydrolysis	99.39 ± 0.01	19.56 ± 0.08	69.64 ± 0.07	19.52 ± 0.08	62.44 ± 0.17
	Two-bath	99.65 ± 0.07		76.54 ± 0.95		70.18 ± 0.86
	One-bath	99.60 ± 0.01		81.37 ± 0.28		71.87 ± 1.24
Green	Alkaline Hydrolysis	88.79 ± 0.60	29.03 ± 1.05	57.37 ± 0.63	28.15 ± 0.97	57.07 ± 0.62
	Two-bath	97.77 ± 0.12		70.89 ± 0.11		66.64 ± 0.00
	One-bath	98.23 ± 0.13		74.26 ± 0.38		73.42 ± 0.61

Table S2. Traces of copper (Cu) from X-ray photoelectron spectroscopy after decolorisation with Na_2CO_3-$Na_2S_2O_4$

Sample	Cu 2p %
Decolorized cotton	0.17 ± 0.04

Table S3. The tensile strength (TS) and tensile strength retention (RTS) results of the four reactive-dyed cotton fabrics after alkaline hydrolysis (Na_2CO_3) color stripping (-SC), two-bath Na_2CO_3□$Na_2S_2O_4$ color stripping (-2), and one-bath Na_2CO_3/$Na_2S_2O_4$ color stripping (-1).

Fabric	Warp		Weft	
	TS (MPa)	R_{TS} (%)	TS (MPa)	R_{TS} (%)
Black (BL)	45.63 ± 0.16	-	43.06 ± 0.53	-
BL-SC	45.59 ± 0.42	99.92 ± 0.91	42.44 ± 0.78	98.56 ± 1.80
BL-2	43.10 ± 0.86	94.46 ± 1.88	41.30 ± 0.95	95.92 ± 2.20
BL-1	45.48 ± 0.34	99.68 ± 0.75	41.49 ± 1.06	96.36 ± 2.46
Grey (GR)	41.12 ± 0.81	-	39.96 ± 0.80	-
GR-SC	39.55 ± 1.03	96.19 ± 2.50	39.80 ± 0.97	99.61 ± 2.44
GR-2	38.91 ± 1.00	94.63 ± 2.43	37.87 ± 0.74	94.79 ± 1.86
GR-1	38.94 ± 0.94	94.69 ± 2.29	37.58 ± 0.96	94.04 ± 2.39
Navy (NV)	62.14 ± 0.97	-	62.46 ± 0.85	-
NV-SC	61.38 ± 0.66	98.78 ± 0.75	61.69 ± 0.97	98.77 ± 1.55
NV-2	60.23 ± 0.93	96.92 ± 1.49	60.86 ± 0.88	97.44 ± 1.40
NV-1	58.64 ± 0.73	94.37 ± 1.18	59.93 ± 0.93	95.95 ± 1.49
Dark Green (DG)	48.84 ± 0.48	-	49.59 ± 0.60	-
DG-SC	47.02 ± 0.37	96.28 ± 0.76	48.78 ± 0.85	98.37 ± 1.71
DG-2	46.66 ± 0.93	95.55 ± 1.55	48.65 ± 0.77	98.10 ± 1.79
DG-1	43.14 ± 1.11	88.34 ± 2.28	47.50 ± 0.72	95.79 ± 1.82

Table S4. Comparison of the results of lab-scale and scaled-up one-bath ($Na_2CO_3/Na_2S_2O_4$) color stripping process performed on different color reactive-dyed cotton fabrics.

Fabric	One-bath process scale	Color stripping %	L*	WI	Weight loss %
Black	Lab-scale	99.59	83.85	80.48	8.07
	Scale-up	99.60	84.03	81.61	7.70
Grey	Lab-scale	97.10	77.64	76.08	5.52
	Scale-up	97.81	79.57	77.33	3.12
Navy	Lab-scale	99.60	81.37	71.87	6.64
	Scale-up	99.55	81.23	73.96	3.32
Dark Green	Lab-scale	98.23	74.26	73.42	6.27
	Scale-up	98.92	77.74	76.81	5.25
